# Oxonium Ion–Guided Optimization of Ion Mobility–Assisted Glycoproteomics on the timsTOF Pro

**DOI:** 10.1016/j.mcpro.2022.100486

**Published:** 2022-12-19

**Authors:** Soumya Mukherjee, Andris Jankevics, Florian Busch, Markus Lubeck, Yang Zou, Gary Kruppa, Albert J.R. Heck, Richard A. Scheltema, Karli R. Reiding

**Affiliations:** 1 Biomolecular Mass Spectrometry and Proteomics, Bijvoet Center for Biomolecular Research and Utrecht Institute for Pharmaceutical Sciences, University of Utrecht, Utrecht, The Netherlands; 2 Netherlands Proteomics Center, Utrecht, The Netherlands; 3 Bruker Daltonik GmbH & Co KG, Bremen, Germany

**Keywords:** *N*-glycopeptides, ion mobility, TIMS, oxonium ions, neutrophils, plasma, ACN, acetonitrile, CAA, chloroacetamide, CCS, collisional cross-section, CE, collision energy, FA, formic acid, FDR, false discovery rate, HRG, histidine-rich glycoprotein, IM, ion mobility, LC, liquid chromatography, lm, linear model, MGF, mascot generic format, MS, mass spectrometry, PASEF, parallel accumulation serial fragmentation, ROI, region of interest, RT, retention time, SCE, stepped collision energy, SDC, sodium deoxycholate, SGP, sialylglycopeptide, TCEP, Tris(2-carboxyethyl)phosphine, TIMS, trapped ion mobility separation, TNAP, tissue nonspecific alkaline phosphatase

## Abstract

Spatial separation of ions in the gas phase, providing information about their size as collisional cross-sections, can readily be achieved through ion mobility. The timsTOF Pro (Bruker Daltonics) series combines a trapped ion mobility device with a quadrupole, collision cell, and a time-of-flight analyzer to enable the analysis of ions at great speed. Here, we show that the timsTOF Pro is capable of physically separating *N*-glycopeptides from nonmodified peptides and producing high-quality fragmentation spectra, both beneficial for glycoproteomics analyses of complex samples. The glycan moieties enlarge the size of glycopeptides compared with nonmodified peptides, yielding a clear cluster in the mobilogram that, next to increased dynamic range from the physical separation of glycopeptides and nonmodified peptides, can be used to make an effective selection filter for directing the mass spectrometer to analytes of interest. We designed an approach where we (1) focused on a region of interest in the ion mobilogram and (2) applied stepped collision energies to obtain informative glycopeptide tandem mass spectra on the timsTOF Pro:glyco-polygon–stepped collision energy-parallel accumulation serial fragmentation. This method was applied to selected glycoproteins, human plasma– and neutrophil-derived glycopeptides. We show that the achieved physical separation in the region of interest allows for improved extraction of information from the samples, even at shorter liquid chromatography gradients of 15 min. We validated our approach on human neutrophil and plasma samples of known makeup, in which we captured the anticipated glycan heterogeneity (paucimannose, phosphomannose, high mannose, hybrid and complex glycans) from plasma and neutrophil samples at the expected abundances. As the method is compatible with off-the-shelve data acquisition routines and data analysis software, it can readily be applied by any laboratory with a timsTOF Pro and is reproducible as demonstrated by a comparison between two laboratories.

## Highlights

• Using ion mobility as a glycoproteomics acquisition routine on the timsTOF Pro.
• Optimization of collisional energies for glycopeptide fragmentation.
• Ion-mobility–based focusing of instrument time on glycopeptides.
• Increasing data quality by combining duplicate spectra generated by instrument speed.
• Glycoproteomics analyses on high-complexity samples (human plasma and neutrophils).

## Graphical Abstract

**Figure:**
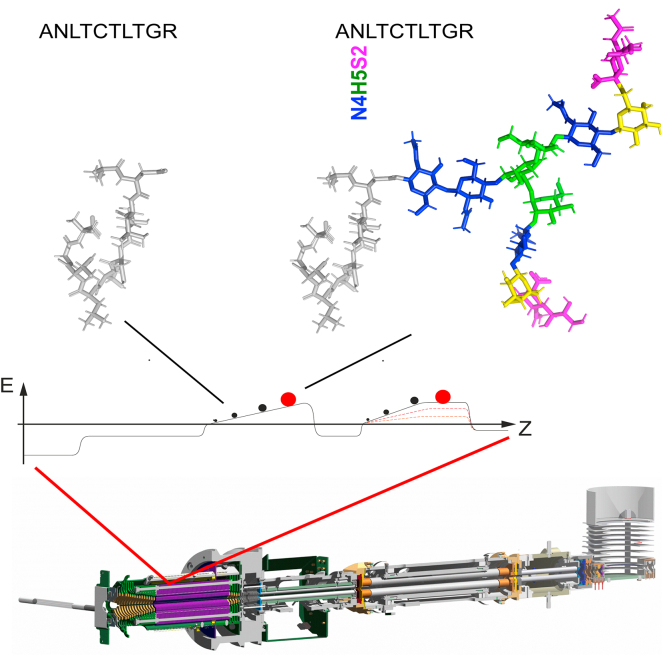

## In Brief

In this study, we advance the use of the timsTOF Pro by designing and applying a novel acquisition routine for glycoproteomics. We demonstrate that the instrument can be specifically focused on sequencing glycopeptides by making use of ion mobility, and that its high scan rate can be used to improve spectrum quality. This can be applied to great effect as we show that, especially for shorter gradients, the number of glycopeptide detections can be sustained at a high level.

Protein glycosylation is a highly abundant co- and post-translational modification, in which glycan moieties of varying complexity are covalently attached to specific residues in proteins (1). Protein glycosylation plays diverse roles in biological systems, influencing processes such as cell–cell adhesion, immunity, and signaling through cellular recognition (2). Glycans most frequently attach to proteins *via* either *N*-glycosidic linkages to asparagine residues (*N*-glycans) or *via O*-glycosidic linkages to the serine or threonine residues (*O*-glycans) (3, 4). A single glycoprotein is known to exhibit multiple glycoforms, displayed by both glycan microheterogeneity (different oligosaccharides can attach at the same site) and macroheterogeneity (glycosylation site occupancy) per site, whereas sites across a given protein can be differentially regulated as well, that is, metaheterogeneity (5). Alterations in these glycosylation patterns have been well documented between physiological and disease states (6, 7). Because of its biological importance and being dynamically regulated in response to any changes in homeostasis, glycosylation is an important target in biomarker research and biopharmaceutical development (8, 9, 10, 11). This emphasizes the need of highly sensitive and precise analytical tools that can identify the highly diverse glycosylation patterns and localize them site-specifically on the proteins they adorn.

Mass spectrometric detection of glycans and intact glycopeptides has emerged as an attractive glycoproteomics analytical platform. Recent progress in workflows, including glycopeptide extraction/enrichment, hybrid mass spectrometric fragmentation, and data analysis, have made detection of glycopeptides increasingly achievable (12, 13, 14, 15, 16, 17, 18). Notwithstanding these advances over the past decade, characterization and quantitation of intact glycopeptides from complex datasets remains a bottleneck because of their inherent glycan heterogeneity, ionization and separation characteristics, and their relative low abundance compared with nonmodified peptide counterparts (19). Optimized methods are clearly needed.

Ion mobility (IM) devices can separate ions by their collisional cross-section (CCS, Ω) at high speed (typically in the order of 10–100 ms) (20, 21, 22). Such devices typically are employed between liquid chromatography (LC) and the mass analyzer to provide an extra level of separation for the molecules of interest and provide improved dynamic range for the mass analysis. For this to work efficiently, a high-speed mass analyzer is required, making time-of-flight analyzers attractive as they can operate at a scan rate in the range of 100 kHz and thus can efficiently sample the ions eluting from the IM. Of the different conceptual devices to achieve gas-phase separation, trapped ion mobility separation (TIMS) can be packaged in a small device only requiring low operating voltages and providing efficient ion usage. In this device, ions are balanced in a constant gas stream by an electrical field allowing them to be stored at different positions. The ions can then be eluted—ordered by low mobility with large CCS to high mobility with small CCS—by lowering the electrical potential after which they are subsequently transferred to the mass analyzer. The timsTOF Pro (Bruker Daltonics) makes this effective combination and was recently shown to provide high analyte coverages in proteomic, lipidomic, and metabolomic studies (23, 24, 25). With the data acquisition approach of parallel accumulation serial fragmentation (PASEF), this instrument is capable to separate and accurately detect biological molecules (peptides, lipids, and metabolites) at very fast scan rates (26, 27).

**Fig. 1:**
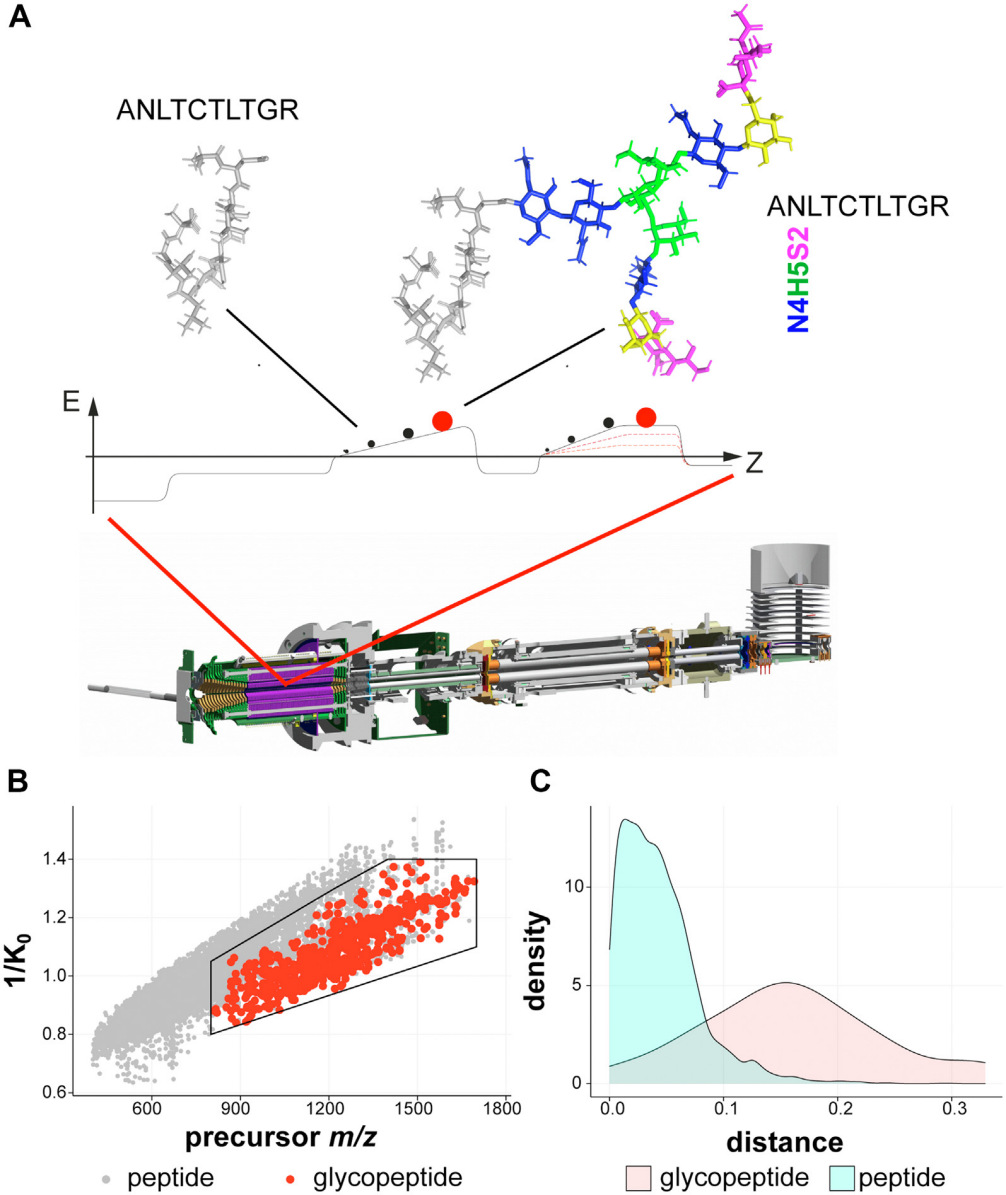
**Ion mobility (IM)–assisted glycoproteomics on the timsTOF Pro.***A*, schematic representation of the instrument with the conceptual operation of the TIMS device separation of the glycopeptides from the nonmodified peptides. *B*, distribution of the ion signals in *m/z versus* IM (1/*K*_0_) for all classes of ions including nonmodified peptides (*gray*) *versus* glycopeptides (*red*). A schematic polygon is shown that encompasses the glycopeptide ion cluster in the IM domain. *C*, density diagram displaying the physical separation of the glycopeptides from unmodified peptides in the IM space (squared distance from linear fit of all data points in the dataset). TIMS, trapped ion mobility separation.

Recently, IM separation mass spectrometry (MS) has emerged as a promising tool for characterizing glycosylated species (28, 29, 30, 31). Glycopeptides, because of their inherent physical properties, have been shown to typically separate from nonmodified peptides within both drift-tube and traveling-wave IM mass spectrometers (32, 33). This enabled glycopeptides to be isolated in individual mobility windows with lower amounts of peptide components and chemical noise. This increases the signal/noise ratio, which is essential for improved detection of low abundant glycopeptide ions. Encouraged by this previous work on the IM separation of glycopeptides from nonmodified peptides (32, 33), we hypothesized that, because of their inherent physical properties, *N*-glycopeptides have different mobility and would therefore cluster in a specific IM region inside the TIMS device that is distinct from nonmodified peptides (Fig. 1). Our aim was to devise a dedicated glycoproteomic workflow on the timsTOF Pro utilizing the region-specific clustering of glycopeptide ions in the TIMS device (glyco-specific polygon), along with optimized glycopeptide fragmentation. In this work, we first optimized the fragmentation settings with two purified glycoproteins for high-quality fragmentation spectra possessing highly visible diagnostic glycan fragments, that is, the glycan oxonium ions, as well as highly informative peptide backbone cleavages that can be used to confidently identify both the peptides and the attached glycan moieties. Optimizing the fragmentation pattern on two simple glycopeptides resulted in a stepped collision energy (SCE) strategy with the PASEF method for optimal glycopeptide fragmentation (SCE-PASEF) for identification and successful sequencing of *N*-glycopeptides. In addition, we demonstrate that the *N*-glycopeptides indeed cluster in a specific IM region that is distinct from the localization of nonmodified peptides, and that physical separation of the two classes of molecules can be achieved (glyco-polygon PASEF). We validated the region of interest (ROI) of glycopeptides in the timsTOF Pro using two biological samples of higher complexity, enzymatic digests of human neutrophils and human plasma, to characterize the IM space occupied by the heterogenous *N*-glycopeptides. Combination of these two strategies (glyco-polygon SCE-PASEF) over the general PASEF method led to a glycoproteomics method capable of identifying diverse and heterogeneous *N*-glycopeptides at both high confidence and high throughput on the timsTOF Pro.

## Experimental Procedures

### Chemicals and Materials

Sodium deoxycholate (SDC), Tris(2-carboxyethyl)phosphine (TCEP), Tris(hydroxymethyl)aminomethane, chloroacetamide (CAA), sodium hydroxide, and TFA were purchased from Sigma–Aldrich. Formic acid (FA) was purchased from Merck. Acetonitrile (ACN) was purchased from Biosolve. Oasis μElution HLB and PRiME HLB plates were purchased from Waters. Milli-Q was produced by an in-house system (Millipore). Both phosphoSTOP and cOmplete Mini EDTA-free were purchased from Roche. GluC was obtained from Roche. Recombinant tissue nonspecific alkaline phosphatase (TNAP) was a gift from Copenhagen Centre of Glycomics. Histidine-rich glycoprotein (HRG) was purified from human plasma with a cobalt-loaded resin (Thermo Scientific) using immobilized metal affinity chromatography–based enrichment (34). Commercial sialylglycopeptide (α2,6-SGP) and asialo-SGP were purchased from Fushimi Pharmaceutical Co, Ltd. Commercial pooled human plasma was purchased from Affinity Biologicals. Purified human neutrophils, prepared as described previously (35), were a kind gift from the Department of Molecular and Cellular Homeostasis, Sanquin Research.

### Proteolytic Digestion of Human Plasma

About 10 μl of pooled nondepleted human plasma was mixed with 50 volumes of SDC buffer (1% SDC and 50 mM Tris–HCl [pH 8.5]), 10 mM TCEP, 30 mM CAA, and boiled for 10 min at 95 °C. The samples were cooled and digested with a combination of Lys-C (1:75 enzyme to protein) for 4 h followed by trypsin (1:20 enzyme to human plasma and 1:35 enzyme to HRG) at 37 °C overnight. The samples were quenched with 10% TFA to a final concentration of 1% TFA (0.5% TFA for HRG), and SDC was precipitated after centrifugation at 14,000 rpm for 10 min. The supernatant was transferred to a new tube and desalted using the μElution HLB plate. The desalted samples were lyophilized and stored at −80 °C before MS analysis.

### Cell Lysis and Proteolytic Digestion of Human Neutrophils

Pooled human neutrophils from healthy donors were enriched by Percoll (GE Healthcare) density gradient centrifugation as previously described (35). Neutrophil cell pellets were resuspended in 100 μl lysis buffer containing 100 mM Tris–HCl (pH 8.5), 7 M urea, 5 mM TCEP, 30 mM CAA, Triton X-100 (1%), 2 mM magnesium sulfate, phosphoSTOP and cOmplete Mini EDTA-free protease inhibitors. Then, cells were disrupted by sonication for 10 min (alternating 20 s off and 40 s off) using a Bioruptor Plus (Diagenode). Cell debris was removed by centrifugation at 14,000 rpm for 1 h at 4 °C, and the supernatant was retained. Impurities were removed by methanol/chloroform protein precipitation as follows: 100 μl of supernatant was mixed with 400 μl of methanol, 100 μl chloroform, and 300 μl of ultrapure water with thorough vortexing after each addition. The mixture was then centrifuged for 10 min at 5000 rpm at room temperature. The upper layer was discarded, and 300 μl of methanol was added. After sonication and centrifugation (5000 rpm, 10 min at room temperature), the solvent was removed, and the precipitate was allowed to dry in air inside a chemical hood. The pellet was resuspended in SDC buffer. GluC was then added to digest proteins for 3 h at an enzyme to protein ratio of 1:75 w/w at 37 °C. The resulting peptide mixtures were further digested overnight at 37 °C by trypsin (1:20 w/w enzyme to protein ratio). The next day SDC was precipitated *via* acidification to a final 0.5% TFA concentration. The peptides in the supernatant were desalted using an Oasis PRiME HLB plate and lyophilized and stored at −80 °C prior to MS analysis.

### Data Acquisition

Tryptic peptides originating from the individual purified glycoproteins, as well as the more complex biological samples, were separated by using an Ultimate 3000 nanoUHPLC (Thermo Fisher Scientific) coupled on-line to a timsTOF Pro mass spectrometer (Bruker Daltonik). Peptides and glycopeptides were analytically separated on an Ion Optics nanoUHPLC column (75 μm × 25 cm, 1.6 μm, C18; Ion Optics) and heated to 50 °C at a flow rate of 400 nl/min. LC mobile phases A and B were water with 0.1% FA (v/v) and ACN with 0.1% FA (v/v), respectively. The nanoLC was coupled to the timsTOF Pro *via* a modified nanoelectrospray ion source (Captive Spray; Bruker Daltonik). Initially, we used a 90 min gradient for the purified glycoprotein samples, whereas the plasma and neutrophil glycoprotein samples were separated using a 150 min gradient. The SGP and asialo-SGP samples were separated using a 15 min gradient. All the LC gradient percentages (% B, time) as well as the short gradients for the plasma samples have been described in supplemental Table S1.

Data acquisition on the timsTOF Pro was performed using OtofControl 6.0 (Bruker Daltonik). Starting from the PASEF method optimized for standard proteomics (26), we integrated the glycan-specific polygon (as depicted in the figures). The following parameters were adapted. For the CaptiveSpray (Bruker Daltonik) source inlet, the capillary voltage was set to 1500 V. The nebulizer dry gas flow rate was set to 3 l/min at 180 °C. TIMS region voltages were optimized at −20, −160, 110, 110, 0, and 75 V for Δ1 to Δ6, respectively. TIMS RF was set to 350 Vpp. The allowed charge states for PASEF precursors were restricted to 2 to 5. The precursor intensity threshold was set to a target value of 20,000 counts, with dynamic exclusion release after 0.4 min. All the MS parameters for the PASEF, glyco-polygon PASEF, SCE-PASEF, and glyco-polygon SCE-PASEF have been described in detail in supplemental Table S2. PASEF without stepping consisted of only one TIMS scan with mobility-dependent collision energy (CE) ramping set at 59 eV from reduced mobility (1/*K*_0_) of 1.60 V s/cm^2^ to 20 eV at 0.6 V s/cm^2^. The collision cell RF (Vpp) was set to 1500 V, and the prepulse storage time was set to 12 μs with 60 μs transfer time. SGP and asialo-SGP were also fragmented with constant CE values starting from 40 to 80 eV in five individual runs with the standard PASEF method. Stepping PASEF MS/MS frame consisted of two merged TIMS scans acquired for low and high CE profile for glycan-specific ions in the SCE (SCE-PASEF) method. CEs (either with stepping or no stepping) were linearly interpolated between the two high and low 1/*K*_0_ values and kept constant above or below these base points (see “Results and discussion” section for more details). The TIMS dimension was calibrated using Agilent ESI LC/MS tuning mix (*m/z*, 1/*K*_0_): (622.0289, 0.9848 Vs/cm^2^), (922.0097, 1.1895 Vs/cm^2^), and (1221.9906, 1.3820 Vs/cm^2^) in positive mode. For filtering glycopeptide-specific PASEF precursors, a modified user-defined polygon filter is described in detail in supplemental Table S2. Human plasma samples were further fragmented using a constant CE value (no linear interpolation of the CE with reduced IM) starting from 40 to 100 eV in seven individual runs with and without the glyco-polygon PASEF method. The efficiency of this specific method was tested using shorter LC gradients (see “Results” section for further discussion).

Plasma and neutrophil measurements were replicated on a second timsTOF Pro instrument at the Bruker (Bremen) laboratory in triplicates, using the four different methods as follows: PASEF, SCE-PASEF, PASEF with glyco-polygon, and SCE-PASEF with glyco-polygon. For the interlaboratory comparison, predigested plasma and neutrophil samples prepared in the first laboratory were aliquoted in duplicate (one for each laboratory) and lyophilized. These lyophilized samples were equivalently resuspended in 2% FA by each laboratory prior to measurement. For analysis, samples were separated on the nanoElute (Bruker Daltonik) coupled on-line to a timsTOF Pro mass spectrometer. Peptides and glycopeptides were analytically separated on an Ion Optics nanoUHPLC column (75 μm × 25 cm, 1.6 μm, C18; Ion Optics) and heated to 50 °C at a flow rate of 400 nl/min. LC mobile phases A and B were water with 0.1% FA (v/v) and ACN with 0.1% FA (v/v), respectively. The nanoLC was coupled to the timsTOF Pro using the CaptiveSpray. All the samples were separated using the same 150 min gradient as used for the previous neutrophil and plasma samples, besides other parameters that were kept the same for comparative analysis between the two laboratories apart from the TIMS region voltages that were set to Δ6 to 55 V and TIMS RF to 450 Vpp (samples used for the interlaboratory comparison are described in supplemental Table S3).

### Data Analysis

The fragmentation spectra from all precursors with charge state >2 were extracted from the recorded Bruker .d format files and stored in mascot generic format (MGF) files with the in-house developed tool HlxlToolchain. The conversion procedure consisted of two steps. In the first step, fragmentation spectra of the same precursor were combined into a single spectrum. Matching of the precursors was performed with the following tolerances: precursor *m/z* ±20 ppm, retention time (RT) ±60 s, and mobility ±5%. Spectral data in “quasi-profile” mode were extracted using the timsDATA 2.17.1.2-beta API obtained from Bruker. Combination of the spectra was achieved by summing peak intensities of all spectra across complete “quasi-profile” *m/z* grid. The final summed spectrum was generated through removal of zero intensity peaks by binning summed “quasi-profile” spectrum in *m/z* bins of 50 ppm. In the second step, each combined spectrum was deisotoped (isotopes were reduced to a single peak at *m/z* of charge state of 1), and TopX filtered at 20 peaks per 100 Th. Together with the conversion procedure, an MGF-meta file was automatically created that contained information on the precursor intensity, mobility (1/*K*_0_), CCS, and monoisotopic mass. The CCS values ware calculated according to the Mason–Schamp equation (36, 37), with the temperature set to 305 K and the molecular weight of N_2_ as the TIMS gas. The MGF files were searched with another in-house tool, HlxlGlyco, which searched specifically for eight glycan–oxonium ions in the MS/MS spectra to preselect the precursors that were likely *N*-glycopeptides: HexNAc, H_14_C_8_N_1_O_5_^+^, 204.0866; Hex-P, H_12_C_6_O_8_P_1_^+^, 243.0264; NeuAc-H_2_O, H_16_C_11_N_1_O_7_^+^, 274.0921; NeuAc, H_18_C_11_N_1_O_8_^+^, 292.1027; Hex-HexNAc, H_24_C_14_N_1_O_10_^+^, 366.1395; Hex-HexNAc-Fuc, H_34_C_20_N_1_O_14_^+^, 512.1974; Hex-Hex-HexNAc, H_34_C_20_N_1_O_15_^+^, 528.1923; and Hex-HexNAc-NeuAc, H_41_C_25_N_2_O_18_^+^, 657.2349. Together with the search, each precursor was associated with a glycan M-score, that is, weighted based on the intensity of the oxonium ions present in the MS/MS spectra, as previously described (38). An oxonium ion meta file was generated containing the information on precursor *m/z*, mobility, CCS, and glycan M-score. The individual CE data files for the human plasma (with and without glyco-polygon) were converted to MGF format, and single combined F2 MGF files were created where all spectra originating from the same precursor using precursor *m/z* ±20 ppm, RT ±60 s, and mobility ±5%, and the intensities were summed together in the final spectrum. The MGF files were searched and processed with MSFragger (version 3.4), FragPipe (version 17.1), IonQuant (version 18.0), and Philosopher (version 4.1.0) for *N*-glycopeptides (17). Briefly, MFG files were searched against the human UniPort FASTA (UP000005640 reviewed with 20,371 entries, downloaded from UniProt on July 30, 2021) with the glyco-N-HCD workflow. Default search parameters were used, where precursor window, lower mass was set to 400 Da, upper mass was set to 5000 Da; precursor and fragment mass tolerance: ±20 ppm; enzyme: full trypsin digestion with two maximum missed cleavages; carbamidomethylation at Cys was set as fixed modification, and oxidation at Met and protein N-term acetylation were set as variable modifications. Peptide filtering at 1% false discovery rate (FDR) was applied through PeptideProphet. Default parameters for *N*-glycan analysis with glycan FDR <1% and glycan mass tolerance 50 ppm were used. The human neutrophil samples were first searched against the same human FASTA file, using Mascot, version 2.7.0.0 (39), using precursor mass tolerance ±20 ppm and fragment mass tolerance ±50 ppm; enzyme: semispecific trypsin + Glu-C digestion; carbamidomethylation at Cys was set as fixed modification, and oxidation at Met and protein N-term acetylation were set as variable modifications. This subsequently yield the top 500 proteins. The human neutrophil MGF files were searched in MSFragger against the top 500 protein database with the glyco-N-HCD workflow, together with semispecific digestion (trypsin-GluC) at a maximum of two missed cleavages at Lys/Arg/Asp/Glu. The output was filtered for the *N*-glycopeptides, and the spectrum scan numbers of annotated spectra were merged with the glycan–oxonium result from HlxlGlyco tool.

Next to MSFragger, the final neutrophil and plasma samples were searched and processed through a combination of MSConvert and Byonic (version 4.4.1, Protein Metrics), in line with previous reports (35, 40), to compare the quantity of post-translational modification occurrences and qualitative glycosylation. Briefly, raw files originating from the timsTOF Pro experiments were converted to the MGF format using MSConvert (3.0.21328-404bcf1), with scanSumming on precursorTol = 0.05, scanTimeTol = 5, and IonMobilityTol = 0.01. The resulting MGF files were searched with Byonic (version 4.4.1), using a list of 279 *N*-glycans set as common1 (35), together with fixed Cys carbamidomethylation and rare Ser/Thr/Tyr phosphorylation, Met/Trp oxidation, and peptide- and protein-N-terminal Glu/Gln pyroglutamic acid formation. Semispecific digestion was allowed with three missed cleavages, at Lys/Arg for the plasma samples and Lys/Arg/Asp/Glu for the neutrophil samples. In alignment with previous studies, peptide-spectrum matches resulting from the Byonic searches were curated to have a score of ≥150 and |log prob| value of ≥1.5.

Further downstream analysis and visual representation of the results was performed with the R(4.03) packages extended with ggplot2 (version 2.3.3.5) and eulerr (version 6.1.1) for data visualization. For visualization of the glycan species, we followed the recommendations of the Consortium for Functional Glycomics (41). Glycan cartoons were constructed and exported from GlycoWorkbench (42).

## Results

### Optimization of the IM ROI for Targeted Analysis of Glycopeptides

**Fig. 2:**
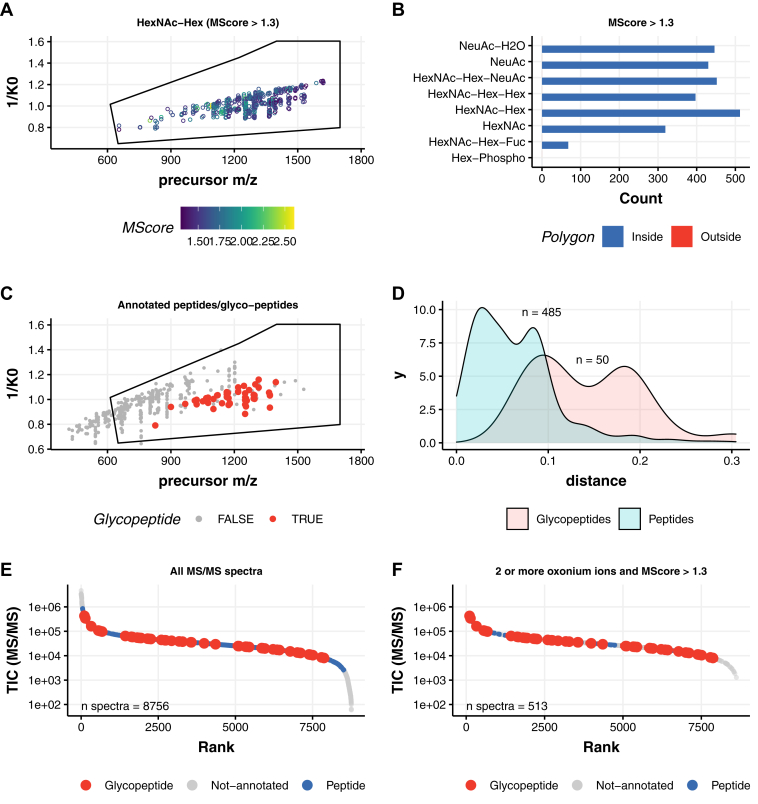
**Glycopeptide identification from the purified tissue-nonspecific alkaline phosphatase (TNAP) protein on timsTOF Pro.***A*, distribution of the precursor ion signals containing *m/z* 366.14 (HexNAc-Hex) oxonium ions, following an M-score cutoff >1.3. *B*, counts of all the glycan diagnostic oxonium ions for TNAP glycopeptides demonstrate localization of all multiply charged *N*-glycopeptide precursors inside the polygon. *C*, distribution of the precursor ion signals in *m/z versus* ion mobility (1/*K*_0_) for annotated peptides and *N*-glycopeptides. *D*, density diagram displaying the physical separation of these annotated peptide species in the mobility space. *E*, ranked distribution of the ion signals for their intensity (TIC [MS/MS]) *versus* rank for all classes of ions (noise in *gray*, annotated nonmodified peptides in *blue*, and glycopeptides in *red*). *F*, ranked distribution of the ion signals following the threshold of at least two oxonium ions and an M-score cutoff >1.3 for the identification of glycopeptide precursor on the ion signals. MS/MS, tandem mass spectrometry; TIC, total ion chromatogram.

We first optimized PASEF data acquisition on purified single glycoproteins guided by the sensitive detection of the diagnostic glycopeptide-derived oxonium ions. Oxonium ions as well as singly charged monosaccharides and oligosaccharides originating from glycopeptide fragmentation were selected as glycopeptide diagnostic species (*m/*z = 204.0872 [HexNAc], *m/*z = 274.0921 [NeuAc-H_2_O], *m/*z = 292.1032 [NeuAc], *m/*z = 366.1400 [HexNAc-Hex], *m/*z = 528.1928 [HexNAc-Hex2], and *m/*z = 657.2354 [HexNAc-Hex-NeuAc]) (supplemental Table S4) to provide a view on the location of the glycopeptides inside the mobilogram of all precursor ions (Fig. 2). Precursor ions with any of these diagnostic ions were observed to cluster inside the IM region comprised of 1/*K*_0_ = 0.8 to 1.4 and *m/z* = 650 to 1700, respectively. To distinguish between chemical noise and oxonium ion containing precursor ion signals, we calculated a weighted oxonium ion score (M-score) that allowed us to select only those MS/MS spectra likely originating from *N*-glycopeptides (38). As expected, most of the MS/MS spectra had an M-score <0.5, as the sample contained also many nonmodified peptides. Previously, it has been suggested that an M-score >1.3 leads to identification of *N*-glycopeptide precursors with an FDR <2.5% (38). Indeed, application of this M-score cutoff led to a selection where almost all the precursors containing at least two oxonium ions were inside the IM polygon (Fig. 2, *A* and *B*). Glycopeptide searches on the data acquired with SCE for the phosphatase TNAP (Fig. 2 and supplemental Figs. S1 and S2) and standard PASEF method for HRG (supplemental Figs. S1,S2 and S4), proteins that have complex glycosylation especially monosialylated and disialylated glycans (N_4_H_5_S_1_, N_4_H_5_S_2_) (supplemental Fig. S1), validated that *N*-glycopeptides are separated in the IM dimension from nonmodified peptides (Fig. 2*C*). To gain insight into this separation, we calculated a linear model (lm) fit for all precursor *m/z* values *versus* mobility in the dataset. The resulting lm equation was used to calculate the Euclidean distances of each annotated peptide to the mobility dimension (*y*-axis). The density plot of the calculated distances demonstrates that precursors generating oxonium ions (annotated glycopeptides) indeed separate from nonmodified peptides (Fig. 2*D*) and could improve the detection of glycopeptides. We in addition plotted the summed MS/MS intensity distribution of all spectra (Fig. 2*E*) and observed a major reduction in chemical noise—from 8756 spectra before filtering to 513 after filtering, with no significant loss in annotated glycopeptides following the application of the M-score cutoff >1.3, with at least two potential oxonium ions in the MS/MS spectra (Fig. 2*F*).

### CE Optimization

As glycopeptides evidently have different gas-phase fragmentation behavior compared with nonmodified peptides, previously optimized settings on the standard PASEF IM–based CE were not optimal to properly fragment *N*-glycopeptides on the timsTOF Pro. Low CEs allow resolving specific glycan structural motifs of *N*-glycopeptides, whereas higher CEs provide information of the site of glycan–protein attachment, peptide fragment ions, and the assignment of features related to glycan core structures such as core-fucosylation (43, 44). SCE-MS/MS combines these two worlds and has been widely used in the high-throughput identification of intact glycopeptides as it generates the most informative and abundant fragment ions for both glycan and peptide sequencing (15). We optimized the CEs for two simple glycopeptides that had different IM (supplemental Figs. S5 and S6). The low energy frame provided diagnostic fragment glyco-oxonium ions (supplemental Fig. S6, *A* and *B*), whereas the high energy frame obtained fragments needed for peptide sequencing. We used these two optimized *N*-glycopeptide fragmentations for linearly extrapolating the calibration curve from reduced IM (1/*K*_0_) 0.6 to 1.6, combining high and low energy frames. This optimized SCE-PASEF method on the timsTOF Pro is crucial for the identifications of the potential *N*-glycopeptides based on the optimal detection of oxonium ions (supplemental Figs. S5 and S6). The resulting curve (supplemental Fig. S6*C*) is sensitive for the detection of specific glyco-oxonium ions and led to the successful identification of 28 unique *N*-glycopeptides originating from the purified protein phosphatase TNAP (Fig. 2).

### Performance on More Complex Samples

**Fig. 3:**
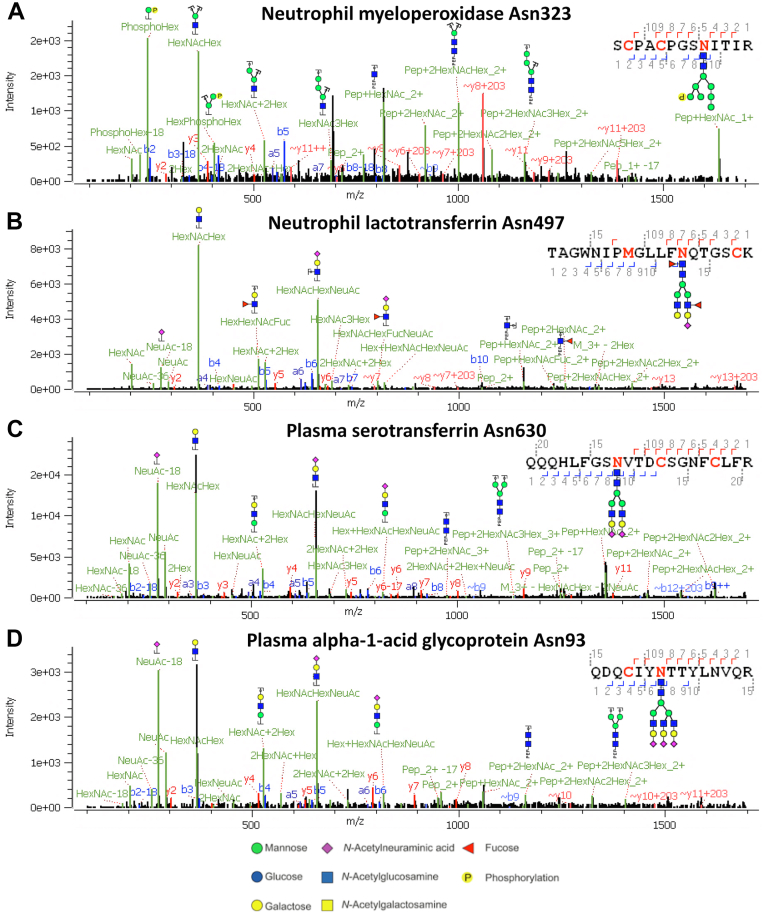
**Illustrative annotated tandem mass spectra of *N*-glycopeptides, displaying the observed diverse glycosylation categories that can be identified using the timsTOF Pro.***A*, phoshomannose glycosylation on a neutrophil myeloperoxidase glycopeptide at Asn323. *B*, antennary fucosylation on a neutrophil lactotransferrin glycopeptide at Asn497. *C*, sialylation on a glycopeptide from plasma serotransferrin at site Asn630. *D*, triantennary species on a glycopeptide from plasma alpha-1-acid glycoprotein at site Asn93. These spectra were obtained by summation of spectra acquired at SCE collision energies. These tandem mass spectra demonstrate the performance of the stepped SCE-MS/MS fragmentation on the timsTOF Pro resulting in glyco-oxonium ions (∼*m/z* 200–700), peptide backbone fragments (*b*- and *y*-ions), and glycan residue losses (B- and Y-fragments). Glycan nomenclature used in glycopeptide definitions is delineated at the bottom of the figure. MS/MS, tandem mass spectrometry; SCE, stepped collision energy.

**Fig. 4:**
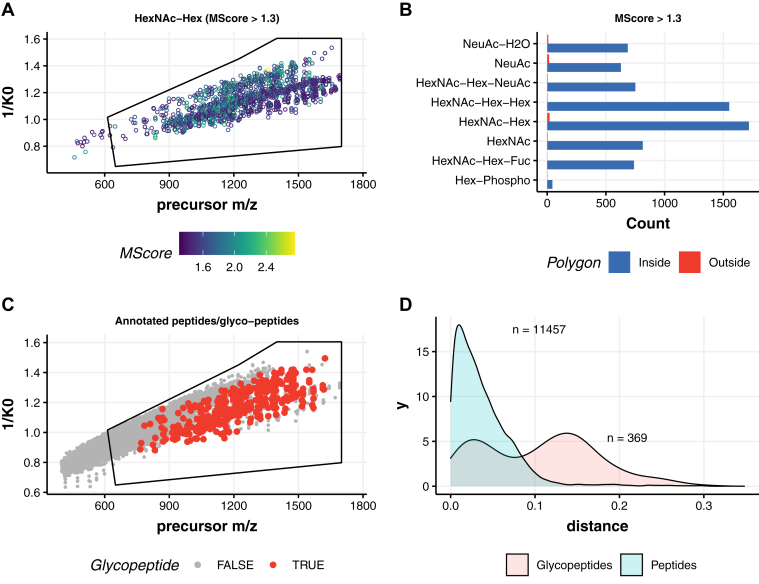
**Identification of *N*-glycopeptides originating from in human neutrophils.***A*, distribution of the precursor ion signals containing *m/z* 366.14 (HexNAc-Hex) oxonium ions, following an M-score cutoff >1.3. *B*, counts of all the glycan diagnostic oxonium ions for neutrophil glycopeptides demonstrate localization of nearly all multiply charged *N*-glycopeptides precursors inside the polygon. *C*, distribution of the precursor ion signals in *m/z versus* ion mobility (1/*K*_0_) for annotated peptides and *N*-glycopeptides. *D*, density diagram displaying the physical separation of these species in the mobility space.

We next subjected (glyco)peptides, derived from neutrophils, postdesalting, to reverse phase-LC-TIMS-MS/MS on the timsTOF Pro with the broad and inclusive polygon and SCE-PASEF fragmentation. First, we evaluated the performance of this glycoproteomic workflow in properly sequencing heterogenous *N*-glycopeptides, including sialylation, fucosylation, as well as pauci-, phospho-, and high-mannose glycans that commonly occur on neutrophil glycoproteins and their resulting glycopeptides (Fig. 3) (35, 40, 45, 46). *N*-glycopeptides originating from the neutrophils (Fig. 4 and supplemental Fig. S7) clustered in a specific region of the IM. Importantly, all high-scoring (M-score >1.3) oxonium ion–containing precursors were clustered inside the polygon (supplemental Fig. S7). The glyco-oxonium ion containing precursors inside the polygon were rather indistinguishable, demonstrating that separation in the TIMS is primarily based on the intact glycopeptide *m/z* and less dependent on the exact nature of the glycan moiety (supplemental Fig. S8). We could identify almost 1500 proteins with a semispecific search at FDR <1%, examples being the abundant neutrophil glycoproteins lactotransferrin and myeloperoxidase. The identified proteome was in congruence with the results from a recent neutrophil proteomics study (47). In our optimized stepped glyco-PASEF method, we identified 440 unique *N*-glycopeptides (222 present in all three replicates) from 54 glycoproteins (supplemental Table S5 and supplemental Fig. S1). In comparison, using a generic PASEF method (without glyco-polygon and SCE) and glyco-polygon PASEF (without SCE), we only detected 244 and 196 *N*-glycopeptides (across three replicates) from 35 and 27 glycoproteins, respectively. In other words, the SCE optimized approach increased the number of identified *N*-glycopeptides across triplicate runs on average by 2.2-fold (supplemental Fig. S9). When visualizing the mobility of the annotated *N*-glycopeptides *versus m/z*, there was a clear physical separation between the glycopeptides and majority of nonmodified peptides (Fig. 4*C*). Using the same lm model calculation approach as described previously and calculating the Euclidean distances for the precursors with M-score >1.3, we observed that the *N*-glycopeptides from the neutrophil samples were also separated from the nonmodified peptides in the IM domain (Fig. 4*D*).

We in addition subjected human plasma to our optimized workflow, using trypsin to digest the proteins. We were able to detect and sequence 518 unique plasma *N*-glycopeptides (275 annotated in all three replicates) originating from 81 unique glycoproteins (supplemental Table S6 and supplemental Fig. S11). In comparison, PASEF and polygon-PASEF (without SCE) methods could identify only 76 and 72 unique *N*-glycopeptides, respectively, from 32 and 31 glycoproteins (supplemental Table S6 and supplemental Fig. S10). This represents a 6.8- to 7.1-fold increase in the identification rate of *N*-glycopeptides when using SCE methods. Of note, SCE-PASEF (without the specific glyco-polygon) performed equally well as stepped glyco-PASEF, where we could identify 526 unique *N*-glycopeptides (288 annotated in all three replicates) from 83 glycoproteins. A total of 67.4% of *N*-glycopeptides overlapped between these two methods with more than 90% overlap at glycoprotein level (supplemental Fig. S12). The lack of benefit is explained by the liberal polygon used to capture all glycopeptides.

**Fig. 5:**
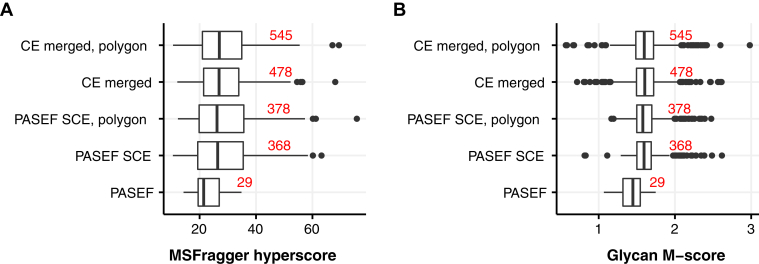
**Performance or glycopeptide annotation using data acquired using PASEF, SCE-PASEF, and SCE-PASEF glyco-polygon methods in comparison to a dataset with merged collision energy (CE) spectra.** Synthetic data files are constructed from data files collected at seven different CEs (40, 50, 60, 70, 80, 90, and 100) measured with (CE merged, polygon) and without (CE merged) glyco-polygon. Numbers in *red* represent count of unique annotated glycopeptides. *A*, clear in peptide annotation score from MSFragger can be observed in SCE data and CE merged results. *B*, application of different CE values significantly improve glycan score of MS/MS spectrum. MS/MS, tandem mass spectrometry; PASEF, parallel accumulation serial fragmentation; SCE, stepped collision energy.

The results from both human neutrophil and human plasma samples, in addition, indicate that to fully exploit the benefits of the glyco-polygon concept, it has to be optimized for specific sample type. In addition, because of the high timsTOF Pro data acquisition speed, it would be possible to use more comprehensive fragmentation methods. For example, as an alternative to SCE method with two predefined CE gradients, each precursor can be measured individually at five or more different CE in separate measurements and then combined to obtain better fragmentation patterns of both the peptide and glycan fragments. As a proof of concept, we collected human plasma data using the standard PASEF method at seven different CEs (40, 50, 60, 70, 80, 90, and 100) with and without glyco-polygon defined. The results (Fig. 5, supplemental Figs. S13, S14 and supplemental Table S1) demonstrate a clear increase in the numbers of annotated glycopeptides, glycan M-score values, and peptide ion coverage (increase in MSFragger hyperscore) in search results where spectra acquired at different CEs are merged into single spectrum. In addition, using the glyco-polygon for data acquisition effectively increased the number of annotated *N*-glycopeptides (545 in CE merge, polygon) by almost 12% in comparison to PASEF method without polygon (478 in CE merge). Thus, in combination with glyco-polygon, this approach provides a further 1.5-fold increment, possibly because of reduction in chemical noise and greater focusing in the ROI on analyte of interest. From these results, it is clear that future developments of the timsTOF Pro methods for glycoproteomics should be aimed toward developing MS/MS methods allowing dynamic application of multiple CEs. To furthermore demonstrate applicability of glycan ion selection polygon, we next focused on shorter chromatography gradients as described in the next paragraph.

### Focusing Leads to Increased Analytical Depth

**Fig. 6:**
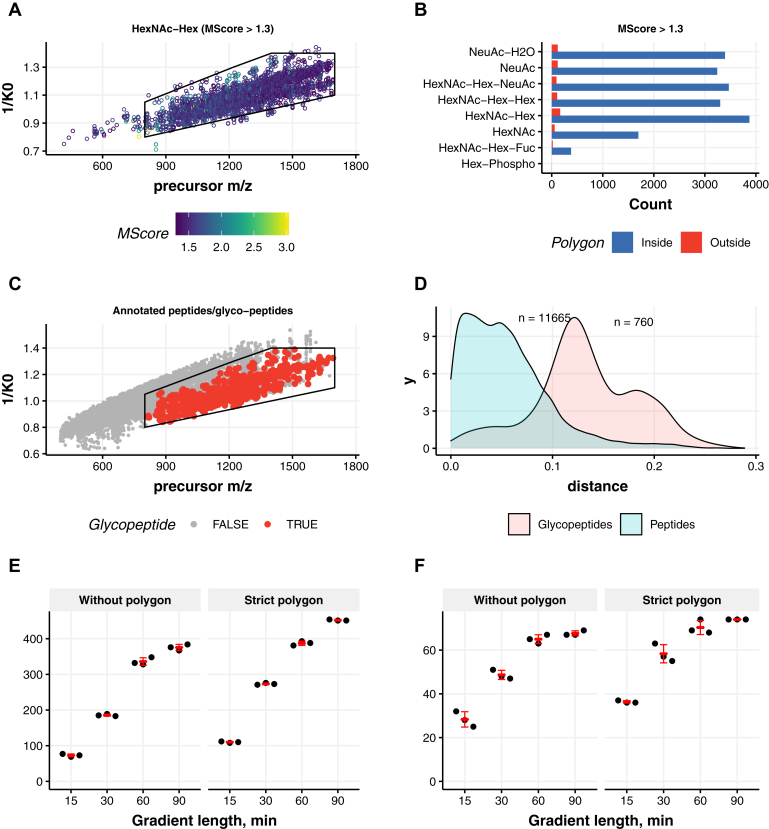
**Identification of *N*-glycopeptides originating from human plasma and high-throughput glycoproteomics.***A*, distribution of the precursor ion signals containing *m/z* 366.14 (HexNAc-Hex) oxonium ions, following an M-score cutoff >1.3. *B*, counts of all the glycan diagnostic oxonium ions for plasma glycopeptides demonstrate localization of all multiply charged *N*-glycopeptide precursors inside the stricter polygon. *C*, distribution of the precursor ion signals in *m/z versus* ion mobility (1/*K*_0_) for annotated peptides and *N*-glycopeptides demonstrate that this smaller polygon contains most of the *N*-glycopeptides, and anything outside this box can be ignored (noisy MS/MS spectra). *D*, density diagram displaying the physical separation of the nonmodified peptides and *N*-glycopeptides in the mobility space is better with this smaller polygon. *E*, unique glycopeptide and (*F*) glycoprotein detection, comparing the more strict polygon with the non–glyco-specific selection (without polygon) for different gradient lengths (15, 30, 60, and 90 min). MS/MS, tandem mass spectrometry.

Having ascertained that the glyco-oxonium ion–containing precursors cluster in a specific ROI, we built a stricter glycopeptide polygon (based on the MSFragger annotations of the glycopeptide-spectrum matches from the broad inclusive glyco-polygon SCE-PASEF results) comprised 1/*K*_0_ 1.05 to 1.4 for *m/z* 800 to 1700, respectively (upper boundary) and 1/*K*_0_ 0.8 to 1.1 for *m/z* 800 to 1700, respectively (lower boundary) (Fig. 6, supplemental Fig. S15), to include only high-scoring and confident *N*-glycopeptides to investigate if there was any advantage of the glyco-specific ROI in IM as well as its fast performance compared with SCE-PASEF method. The sensitivity and efficiency of the method was tested using sequentially shorter gradient runs on human plasma sample. For the same plasma sample, we identified 452 unique *N*-glycopeptides (mean across three replicates) from 74 glycoproteins using the polygon method compared with 376 unique *N*-glycopeptides from 67 proteins using the nonpolygon method (Fig. 6, *E–F*). As expected, the new method retained better performance in subsequently shorter gradients as well (Fig. 6, *E–F* and supplemental Table S8), the largest difference presenting itself at a 30 min gradient with the detection of approximately 1.5-fold more unique *N*-glycopeptides when the strict polygon was used. As the complexity and dynamic range of mass spectrometers are expected to increase further in the coming years, this indicates that the polygon (*i.e.*, focused) method will provide superior performance.

We investigated whether in-source, or rather in-TIMS, water losses would be feasible candidates for better glycopeptide annotation as a significant increase in annotations has reported previously (48). For every precursor mass from the SCE-PASEF 90 min gradient data with and without polygon, it was verified whether an accurate mass could be matched to a water loss (−18.0100) mass difference using a 20 ppm mass window and RT window of 20 s. When looking at precursor intensity and total MS/MS intensity, we observed that these were consistently higher in “parent” precursor than in the matching potential water-loss precursors (supplemental Fig. S16, *A* and *B*). In addition, only 24 potential water-loss ions from almost 30,000 precursors present in the data file could be found in SCE-PASEF polygon data file. This suggests that in-source water-loss fragmentation is more abundant in unmodified peptides than in glycopeptides. A relatively small number of the selected precursors matched the M-score filter criteria (supplemental Fig. S16*C*). Although a slight increase in M-score was found for a few potential water-loss precursors, the downside of adding water loss to the search parameters is that the search space is expanded leading to lower numbers of identified glycopeptides.

### Qualitative Comparison of Peptide Glycoforms

**Fig. 7:**
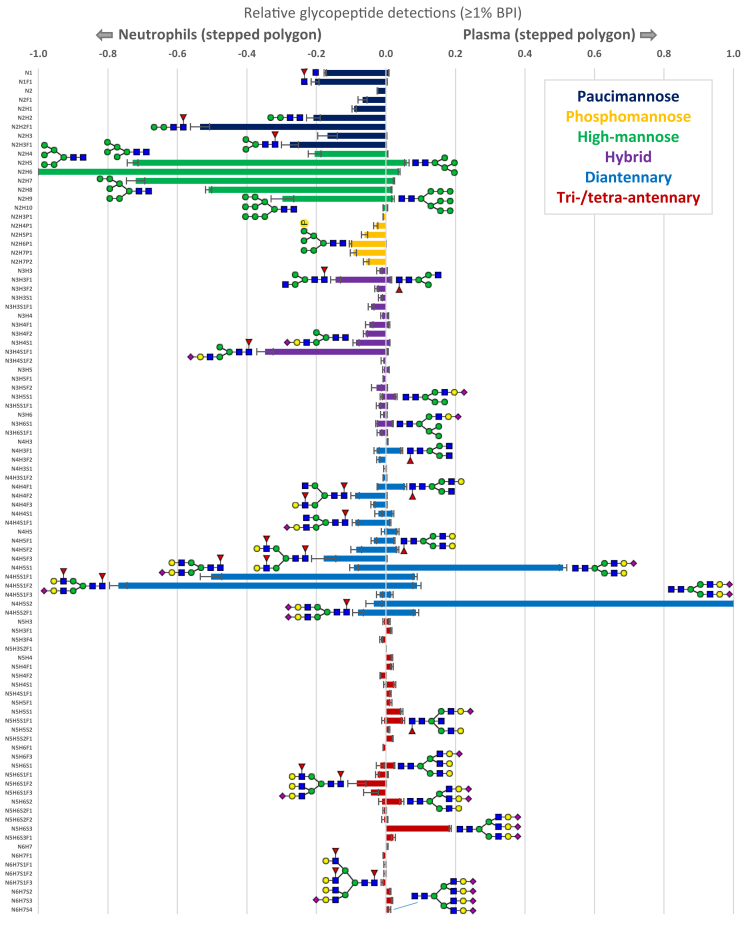
**Qualitative comparison of peptide glycan repertoires observed in human neutrophils (*left*) and human plasma (*right*).** Glycan species were included in the overview when present in at least 1% of relative peak abundance in any of the six samples (3× plasma, 3× neutrophil). Error bars represent the standard deviation for the relative quantification across triplicate injections. We assigned global glycosylation characteristics on the glycopeptides based on the monosaccharide composition, and for visualization purposes, we defined the following glycosylation characteristics: (1) paucimannose (HexNAc <3 and Hex <4), (2) phosphomannose (phospho >0), (3) high-mannose (HexNAc = 2 and Hex >3), (4) hybrid/asymmetric (HexNAc = 3), (5) diantennary (HexNAc = 4), and (6) extended (HexNAc >4) (35).

Finally, we qualitatively compared the peptide glycoforms from the two complex biological samples (Fig. 7). Using the glyco-polygon SCE-PASEF method results on the plasma glycoproteome, we observed that several *N*-glycan compositions dominated, in line with previous reports (49). The glycan repertoires included diantennary and triantennary glycan species, with varying degrees of sialylation, that largely originate from liver-produced acute phase proteins such as haptoglobin, α-2-HS-glycoprotein, and α-1-acid glycoprotein, partially galactosylated glycans that are mainly found on the varying subclasses of immunoglobulin G, as well as high-mannose glycans stemming from proteins like immunoglobulin M, apolipoprotein B-100, and complement C3 (49). The glyco-polygon SCE-PASEF data of the neutrophil samples, on the other hand, distinctly showed phospho- and paucimannose glycans (and smaller) occurring on azurophilic granule proteins like myeloperoxidase, proteinase 3, and cathepsin G, highly fucosylated complex glycans on, for example, lactotransferrin and neutrophil gelatinase–associated lipocalin, as well as high-mannose species on membrane-anchored proteins like integrin alpha-M and integrin beta-2. Again, these detections were highly consistent with what was previously reported for the same sample type, yet with different MS instrumentation (35).

### Interlaboratory Comparison of the Optimized Oxonium Ion–Guided IM-Assisted Glycoproteomics Workflow on the timsTOF Pro

**Fig. 8:**
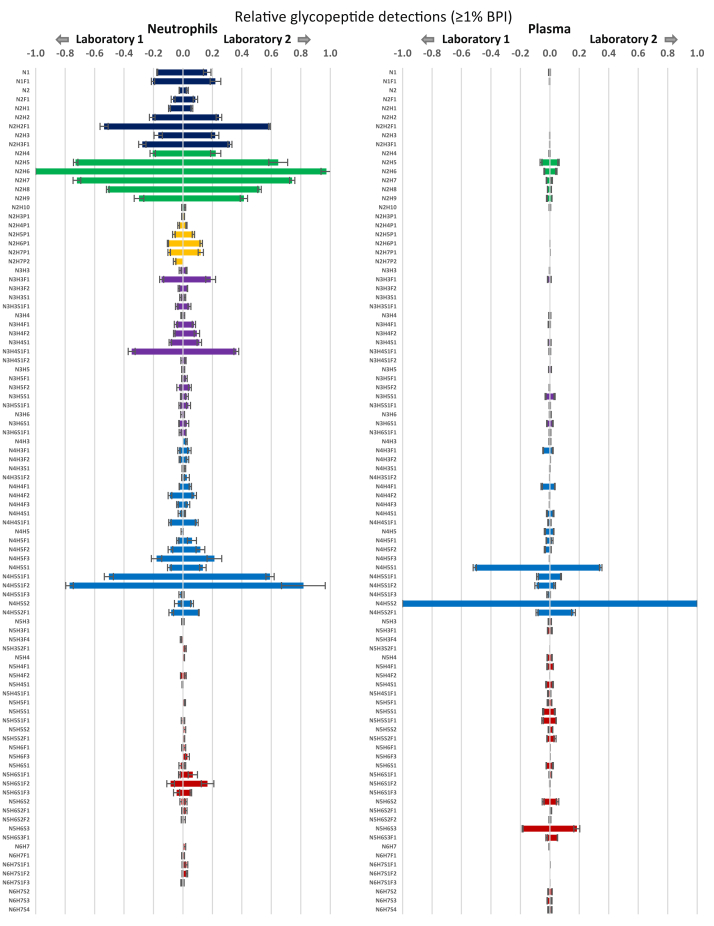
**Interlaboratory comparison of the detected peptide glycan repertoires observed in human neutrophils (*left*) and human plasma (*right*).** Error bars represent the standard deviation for the relative quantification across triplicate injections. Using the same samples and optimized workflow laboratory 1 and 2, respectively, detected 321 and 281 glycopeptides in neutrophils and 389 and 452 glycopeptides in plasma.

Proteomics and, possibly even more so, glycoproteomics experiments are often hampered by limited reproducibility, especially when comparing data obtained between different laboratories with different workflows for data acquisition and analysis (50, 51). To test the robustness of the method presented here, we transferred digested human plasmas and neutrophils to a second laboratory with independent operators—aliquots of the same samples that were used for method optimization. Using the optimized SCE-PASEF method with glyco-polygon, results from the second laboratory yielded ∼60% overlap for the reproducibly detected *N*-glycopeptides (present in all three replicates) in neutrophil and plasma samples (supplemental Fig. S17; supplemental Tables S5 and S6). In addition, the number and identity of the unique glycopeptides were very much alike as well, as illustrated by the highly similar distributions of glycan moieties (Fig. 8). The most notable differences in reproducibility were the absence of N_2_H_7_P_2_ in the second measurement as well as relatively higher numbers of detections in already highly abundant species, for example, N_4_H_5_S_2_F_1_ (0.08 *versus* 0.11) and N_4_H_5_S_1_F_2_ (0.77 *versus* 0.82) in the neutrophil sample and N_4_H_5_S_2_F_1_ (0.09 *versus* 0.16) and N_5_H_6_S_3_F_1_ (0.02 *versus* 0.05) in the plasma sample. This may still denote differences in sensitivity or linear range between measurements or may be due to sample transport. In general, however, congruence between samples was very high (*R*^2^ = 0.9958 on the dot product).

## Discussion

In this study, we used a nanoflow glycoproteomics workflow utilizing the advantages of both the TIMS and PASEF methods on the timsTOF Pro. Our results indicate that physical separation can be achieved for *N*-glycopeptides in the TIMS compared with nonmodified peptides, both when starting from purified single glycoproteins as well as more complex and diverse samples such as plasma. This separation helps to increase the analytical depth, which will be beneficial for future glycoproteomic analyses. A dedicated glycan-specific polygon in the PASEF mode, together with SCE, significantly enhanced the *N*-glycopeptide identification by effectively increasing spectrum quality and maximizing the time spent on specific analytes of interest. The glyco-polygon SCE-PASEF provided an almost 10-fold increase compared with the original PASEF method (Fig. 5). This resulted in a significant increment in the identifications of *N*-glycopeptides in all samples studied but especially so for shorter gradients (Fig. 6). We could identify >300 unique *N*-glycopeptides from human neutrophils and >400 unique *N*-glycopeptides from plasma, resulting in a 2.2- and 7-fold increase, respectively, compared with the standard PASEF method (150 min RT gradient). Recently, 352 unique *N*-glycopeptides (89 glycoproteins) (38) have been identified in plasma (undepleted), which was comparable with our SCE-PASEF results (452 *N*-glycopeptides and 74 glycoproteins). Of note, our merged CE with glyco-polygon resulted in >560 *N*-glycopeptides demonstrating better performance compared with affinity-based glycoproteomic workflows (478 *N*-glycopeptides) on human plasma (52). Interestingly, application of the glyco-polygon with SCE-PASEF consistently outperformed SCE-PASEF alone, enabling either faster analysis through shorter gradients or increased analytical depth for longer gradients. Especially the first makes our workflow very attractive for glycoprotein biomarker diagnostics when larger cohorts are assessed.

However, our method also has still some drawbacks. The oxonium ions that are typically used to differentiate glycan isomers on other types of mass spectrometers fall outside the lower mass range of our instrumental setup, effectively preventing detection of anything smaller than a HexNAc (∼*m/z* 204). Extending the mass range toward lower *m/z* values to include HexNAc fragments and hexose oxonium ions would not only help detections in general but also present the opportunity to distinguish some glycan isomerism (GlcNAc *versus* GalNAc) (53, 54). An attractive strategy for the future would be to also be able to combine glycan structure or isomer detection using IM separations.

The glycosylation characteristics we ultimately observed for the neutrophil and plasma samples proved to be highly congruous with earlier reports employing different instrumentation and methods (35, 49, 55). Neutrophil digests are especially challenging because of the high abundance of very small glycan species (paucimannose and smaller), labile phosphomannose residues, as well as large glycans with a complex pattern of sialylation and fucosylation on their glycopeptides (35, 40, 45, 46). Nevertheless, all these characteristics proved recoverable within our experiments, and while running the timsTOF Pro with standard PASEF led to a noticeable undersampling of the more complex glycans, the detection of glycopeptides across the full complexity space was allowed by the application of SCE and polygon selection. Interestingly, in the comparison between neutrophils and plasma, it was noted that sialylation (high in plasma) and fucosylation (high in neutrophils) were remarkably well assigned according to the literature expectations, even while using the same search parameters. The Fuc2 and Sia1 distinction is a pervasive analytical challenge in MS, as these only differ by 1 Da, and are therefore easily coisolated for fragmentation and/or misassigned in data analysis pipelines. Nevertheless, it must be noted that the glycosylation characteristics we included in our searches do not constitute an exhaustive list. Depending on the biological source, additional glycan features may include sulfation, sialic acid acetylation, GlcNAc sialylation, di-/polysialic acid, NeuGc, and others, and we have thus far limited the size and scope of the glycans to roughly tetra-antennary. We expect that the inclusion of the selection polygon will need to be verified, and possibly adjusted, for the features we have not yet covered, but the here-provided methodology should assist with that. We did include the relatively uncommon phosphomannosylation as this is an abundant hallmark of neutrophil proteins, as shown by prior neutrophil glycoproteomics (35), glycoproteomics of isolated neutrophil proteins (40), as well as by earlier histochemical methods using M6PR-biotin/streptavidin-gold staining (56).

Altogether, based on our observations, we recommend the use of the glyco-polygon SCE-PASEF acquisition method as glycoproteomics workflow on the timsTOF Pro. Given this acquisition method, a 30 min gradient is ideal for sequencing glycoproteins, whereas for complex samples, a 90 min gradient seems more optimal. While it is still challenging for any MS method to ascertain what the unbiased “true” glycosylation profile is of any complex sample, we here report a powerful new means for glycoproteomics that is rapid, broad, and deep. We envision the use of IM-assisted glycoproteomics for future clinical cohort studies and biomarker development as well as for rapid clinical screening to achieve patient stratification.

## Data Availability

The MS proteomics data have been deposited to the ProteomeXchange Consortium *via* the PRIDE (57) partner repository with the dataset identifier PXD034845.

## Supplemental Data

This article contains supplemental data.

## Conflict of interest

F. B., M. L., and G. K. are employees of Bruker Daltonik GmbH, manufacturer of the timsTOF Pro, and thus declare no competing interests. All other authors declare no competing interests.
