# Supplementary material for: Oxonium Ion–Guided Optimization of Ion Mobility–Assisted Glycoproteomics on the timsTOF Pro
Source: Mol Cell Proteomics. 2022 Dec 19;22(2):100486. doi: 10.1016/j.mcpro.2022.100486 (PMC9853368; doi:10.1016/j.mcpro.2022.100486)
Supplement: Supplemental Figures S1–S17 and Table S1–S8 [file mmc1.docx]

**Oxonium Ion-Guided Optimization of Ion Mobility-Assisted Glycoproteomics on the timsTOF Pro**

Soumya Mukherjee^1,2*†^, Andris Jankevics^1,2*^, Florian Busch^3^, Markus Lubeck^3^, Yang Zou^1,2^, Gary Kruppa^3^, Albert J. R. Heck^1,2^, Richard A. Scheltema^1,2$^, Karli R. Reiding^1,2$^

^1^Biomolecular Mass Spectrometry and Proteomics, Bijvoet Center for Biomolecular Research and Utrecht Institute for Pharmaceutical Sciences, University of Utrecht, Padualaan 8, 3584 CH Utrecht, The Netherlands;

^2^Netherlands Proteomics Center, Padualaan 8, 3584 CH Utrecht, The Netherlands;

^3^Bruker Daltonik GmbH, Fahrenheitstrasse 4, 28359 Bremen, Germany

^*^These authors contributed equally

^$^Corresponding authors: [r.a.scheltema@uu.nl](mailto:r.a.scheltema@uu.nl) & [k.r.reiding@uu.nl](mailto:k.r.reiding@uu.nl)

†Current Address: Department of Neurology, Washington University at St Louis, 660 Euclid Avenue, St Louis, MO, 63110, USA.


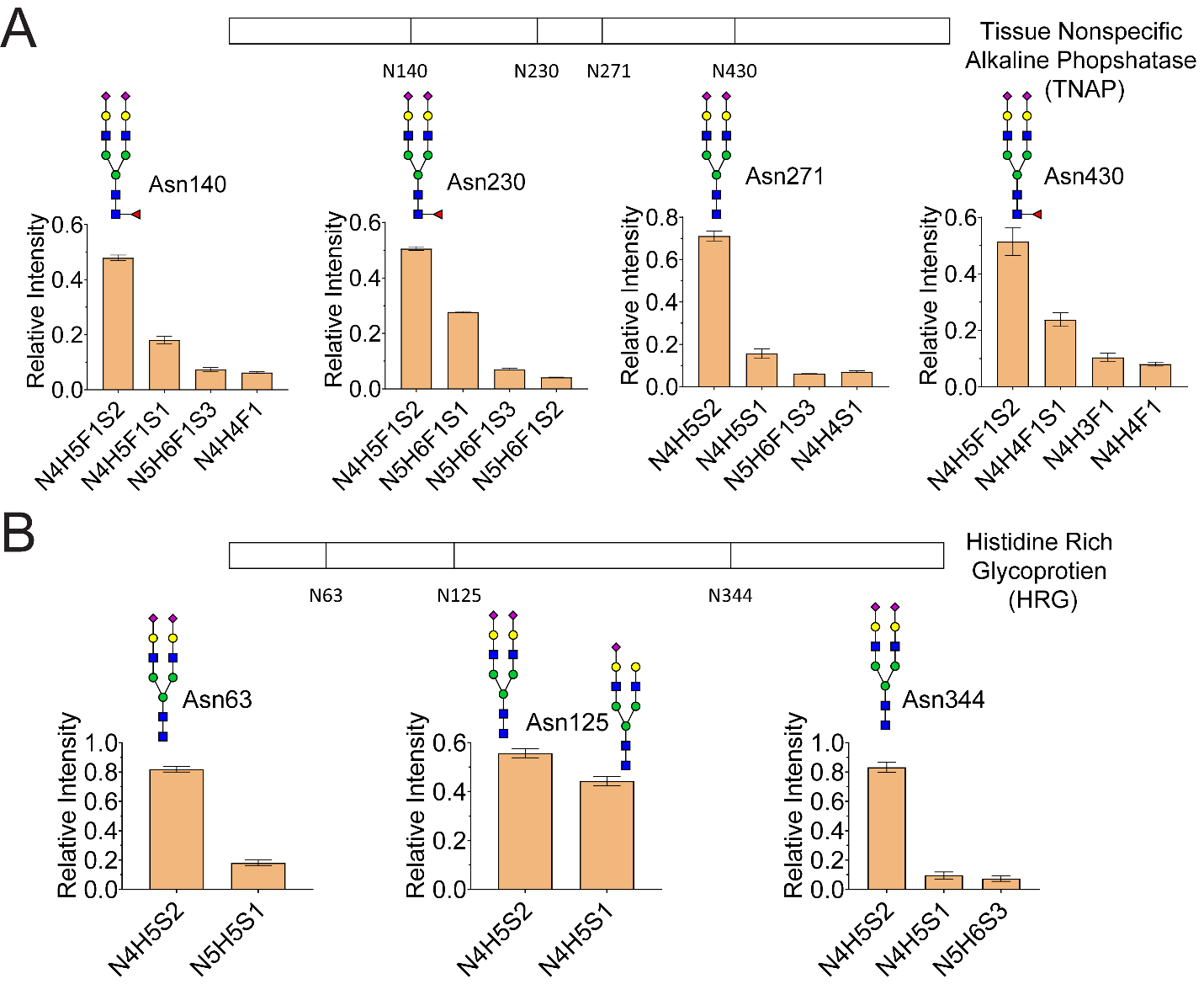


**Figure S1.** Glycan heterogeneity observed in the **(A)** 4 glycosites in the tissue nonspecific alkaline phosphatase (TNAP) and **(B)** 3 glycosites in the histidine rich glycoprotein (HRG) from human plasma. Skyline v21.1.0.146 was used to process the glycopeptides using the top three isotopic peaks from precursor charge states +2 and +3. Integrated peak areas of each glycopeptide were summed up to represent the abundance and was used to estimate the relative abundances.


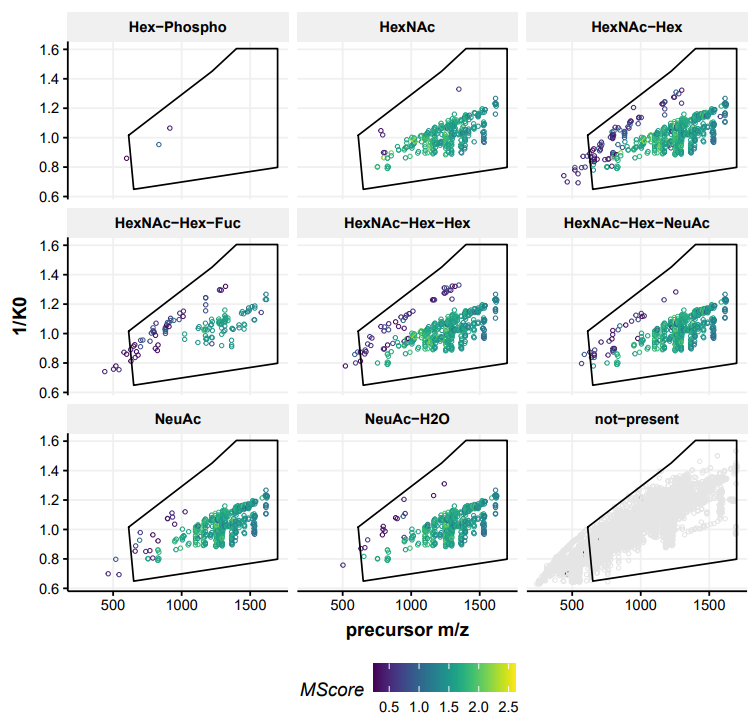


**Figure S2.** Density plot for the distribution for *m/z* vs reduced ion mobility (1/k_0_) of all the precursor ions containing the glyco-oxonium ions 243. 0270 (Hex-Phospho), 204.0872 (HexNAc), 366.1400 (HexNAc-Hex), 512.198 (HexNAc-Hex-Fuc), 528.198 (HexNAc-Hex-Hex), 657.2354 (HexNAc-Hex-NeuAc), 292.1032 (NeuAc) and 274.0921 (NeuAc-H_2_O) as observed for the TNAP protein. All glycopeptide precursors with M-score > 0.5 can be contained within ion selection polygon.


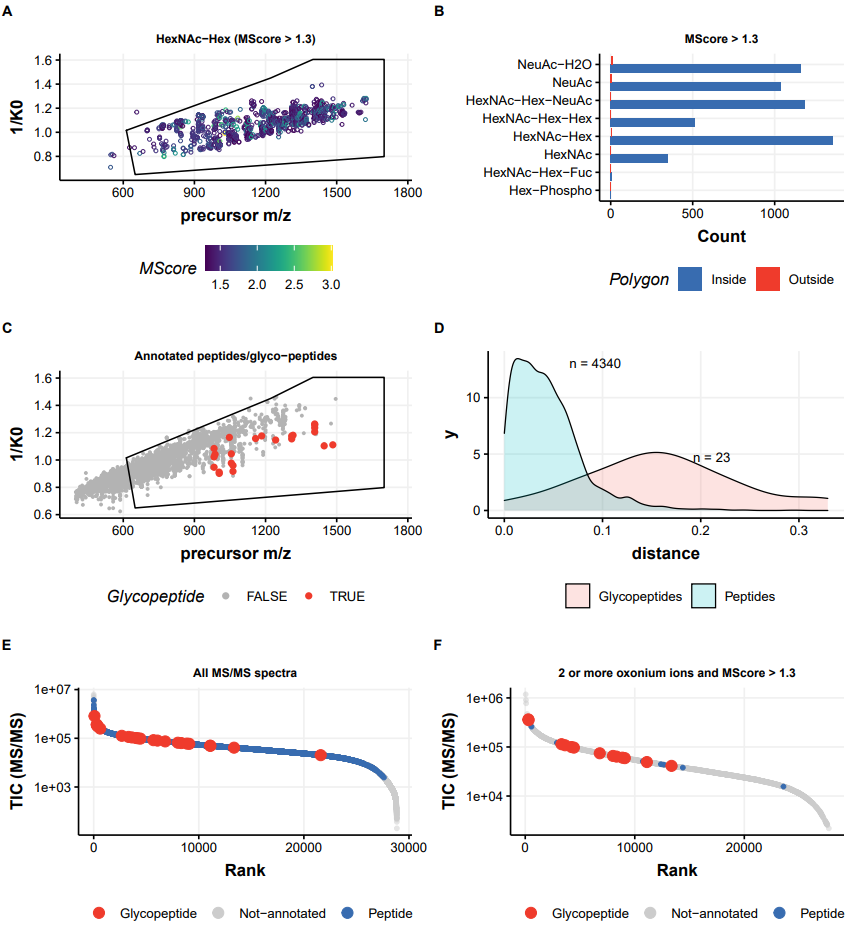


**Figure S3.** Glycopeptide identification from the plasma purified histidine rich glycoprotein (HRG) on the timsTOF Pro. Similar to Figure 2, **(A)** Distribution of the precursor ion signals containing *m/z* 366.14 (HexNAc-Hex) oxonium ions, with a MScore cut-off > 1.3. **(B)** Counts of all the glycan diagnostic oxonium ions for HRG glycopeptides demonstrate localization of all multiply charged *N*-glycopeptides precursors inside the polygon. **(C)** Distribution of the precursor ion signals in *m/z* vs ion mobility (1/K_0_) for annotated peptides and *N*-glycopeptides and **(D)** Physical separation of these species in the mobility space. **(E)** Ranked distribution of the ion signals for their intensity (TIC(MS/MS) vs Rank for all classes of ions (noise, annotated peptides and glycopeptides) and **(F)** Same distribution following the application of 2 or more oxonium ions and MScore cut-off > 1.3 for identification of glycopeptide precursor on the ion signals. The legend for the color-coding is provided at the bottom of the figure.


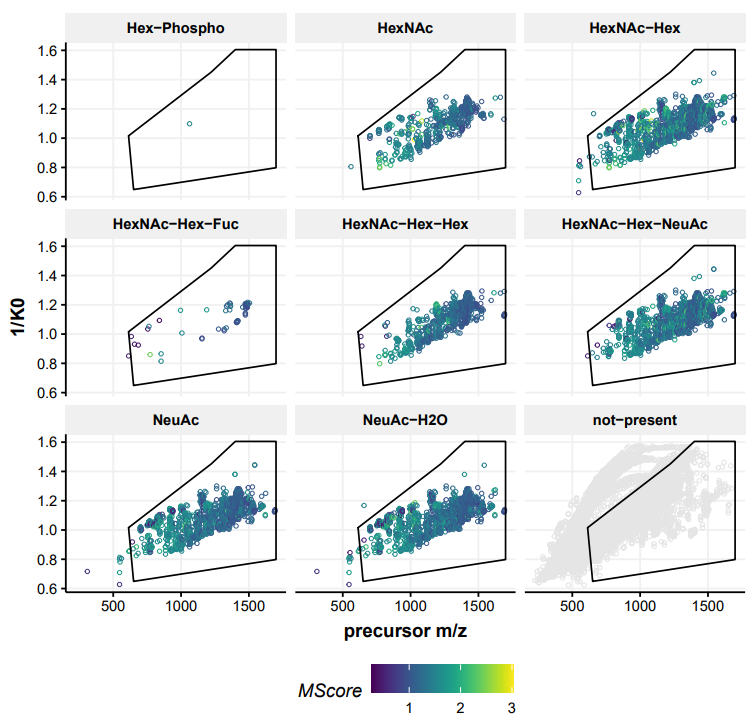


**Figure S4.** Density plot for the distribution for *m/z* vs reduced ion mobility (1/k_0_) of all the precursor ions containing the glyco-oxonium ions 243. 0270 (Hex-Phospho), 204.0872 (HexNAc), 366.1400 (HexNAc-Hex), 512.198 (HexNAc-Hex-Fuc), 528.198 (HexNAc-Hex-Hex), 657.2354 (HexNAc-Hex-NeuAc), 292.1032 (NeuAc) and 274.0921 (NeuAc-H_2_O) for the plasma purified HRG protein. The black polygon contains the glycopeptide precursor with M-score > 1.3.


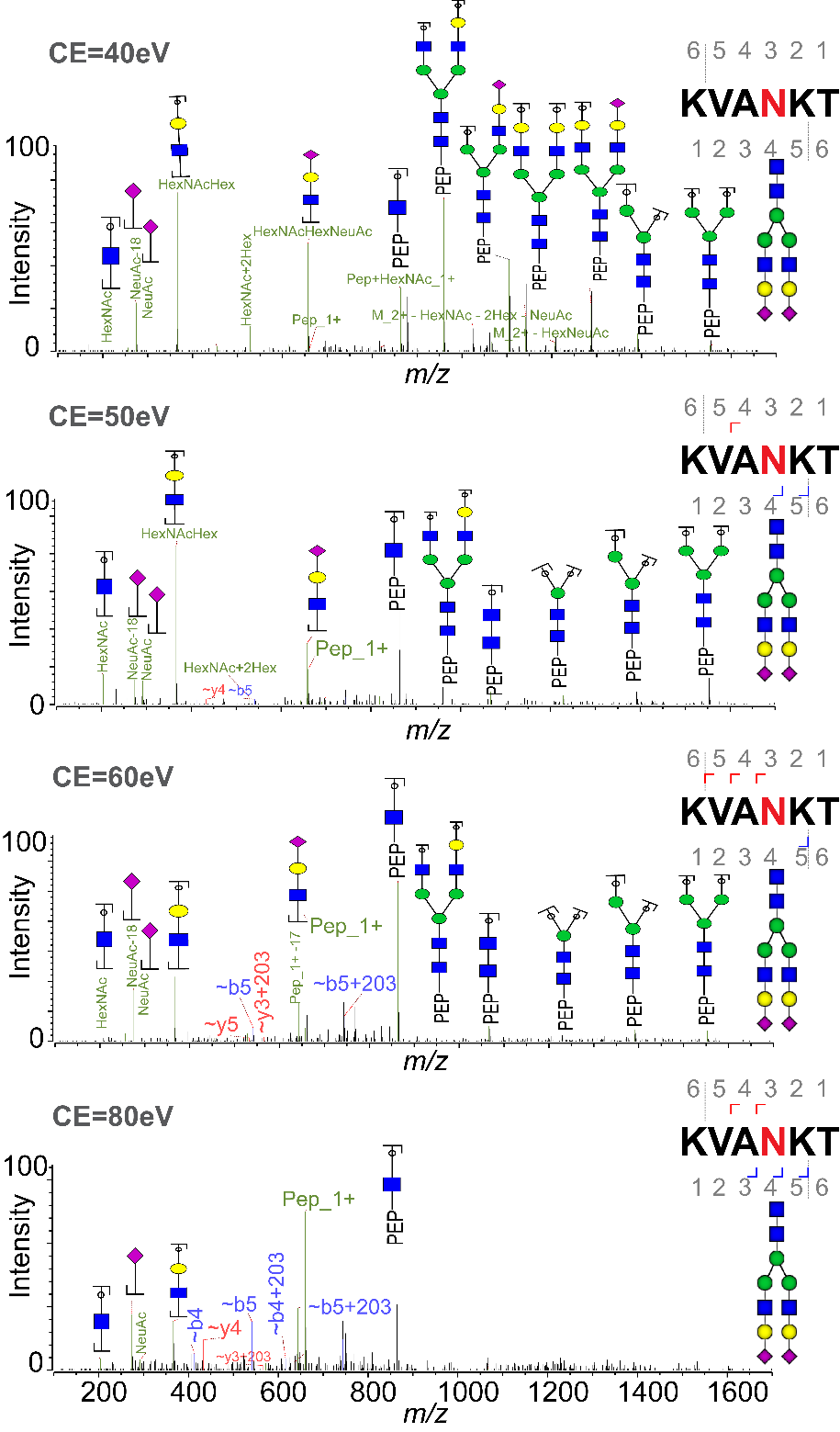


**Figure S5.** Fragmentation spectra for the SGP (α2,6-SGP) at charge state 3 at different collision energies on timsTOF Pro. At low collision energies, Y ions (glycan neutral loss) predominates, while peptide ions (b and y ions) start to appear at elevated energies. At energies higher than the optimal, glycopeptide containing ions start to disappear due to overfragmentation along with the loss of diagnostic oxonium ions.

**
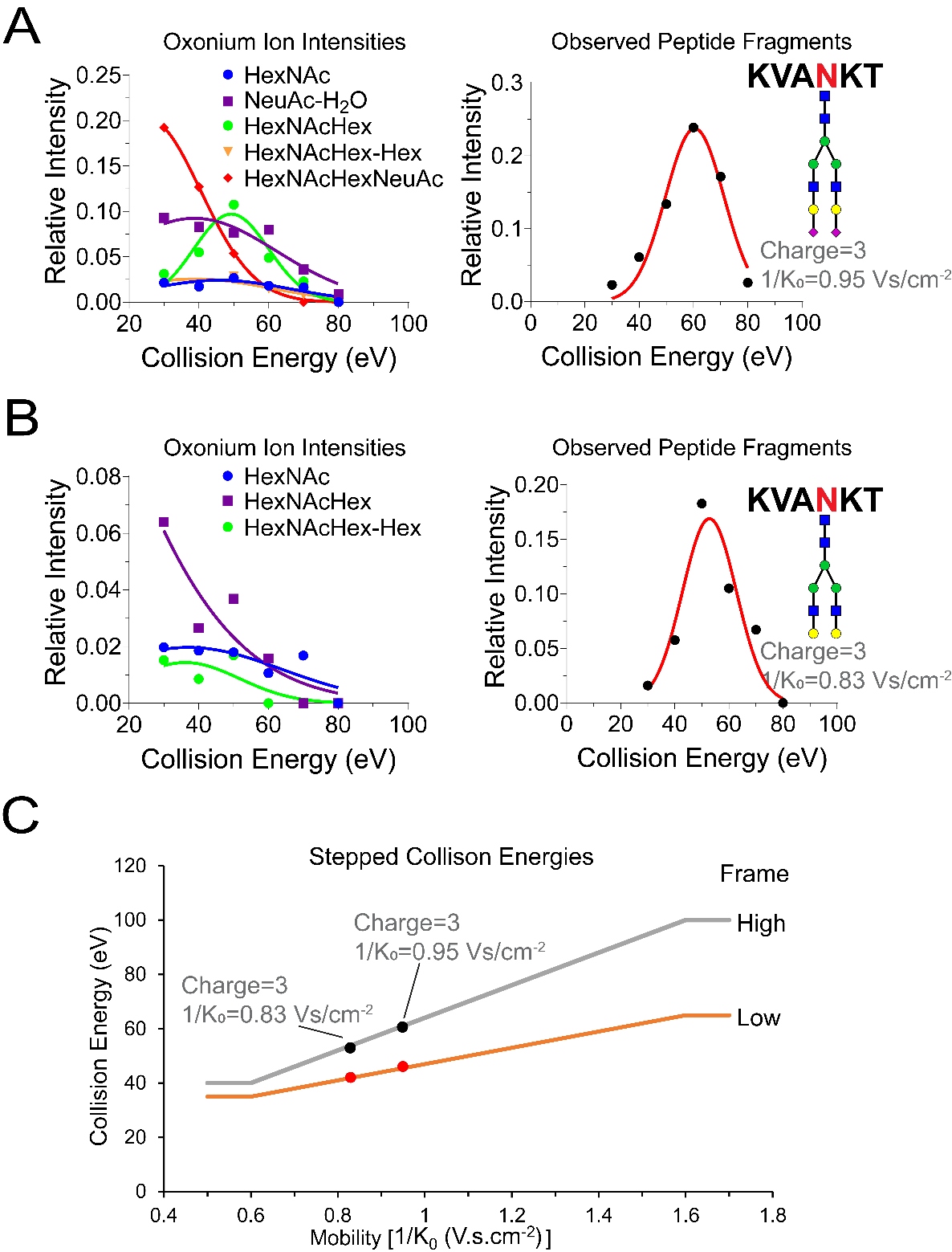
**

**Figure S6.** Optimized stepped collision energies for optimal glycopeptide detection on timsTOF Pro. Relative intensities of the oxonium ions and peptide fragments (b and y ions) at various collision energies for **(A)** SGP at reduced ion mobility (1/K_0_) 0.95 for charge state 3 and **(B)** asialo-SGP at reduced ion mobility (1/K_0_) 0.83 for charge state 3. The optimal collision energy for SGP peptide fragments is at ~ 60 eV and for asialo-SGP at ~ 50 eV, while the optimal collision energies for oxonium ions were ~ 45 and ~ 40 eV, respectively. **(C)** Final stepped collision energies (SCE) calibration curve linearly extrapolated based on these two glycopeptides from reduced ion mobility (1/k_0_) 0.6 to 1.6, combining high and low energy frames.


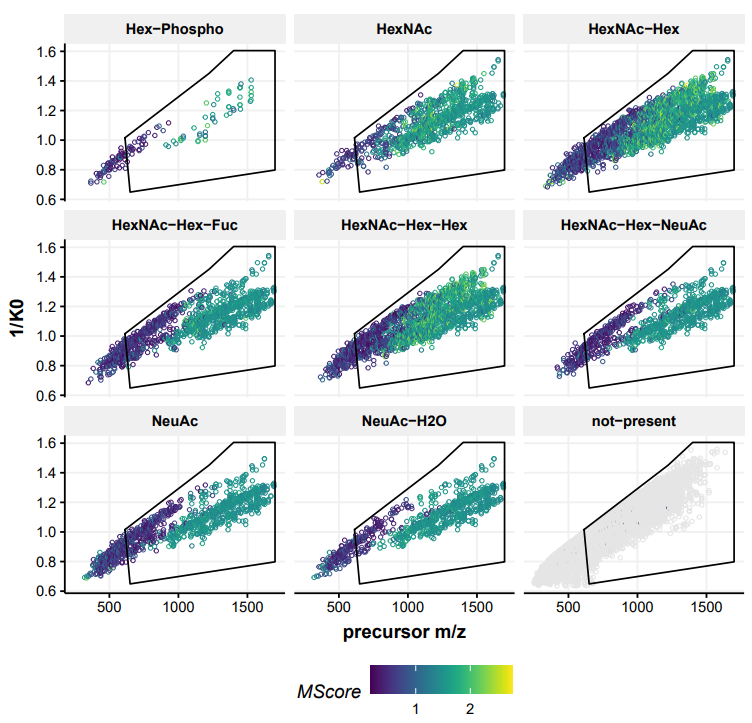


**Figure S7.** Density plot for the distribution for *m/z* vs reduced ion mobility (1/k_0_) of all the precursor ions containing the glyco-oxonium ions 243. 0270 (Hex-Phospho), 204.0872 (HexNAc), 366.1400 (HexNAc-Hex), 512.198 (HexNAc-Hex-Fuc), 528.198 (HexNAc-Hex-Hex), 657.2354 (HexNAc-Hex-NeuAc), 292.1032 (NeuAc) and 274.0921 (NeuAc-H_2_O) from human neutrophils. While this black polygon contains all the glycopeptide precursor with M-score > 1.3, precursors with M-score < 1.3 are mostly noise.


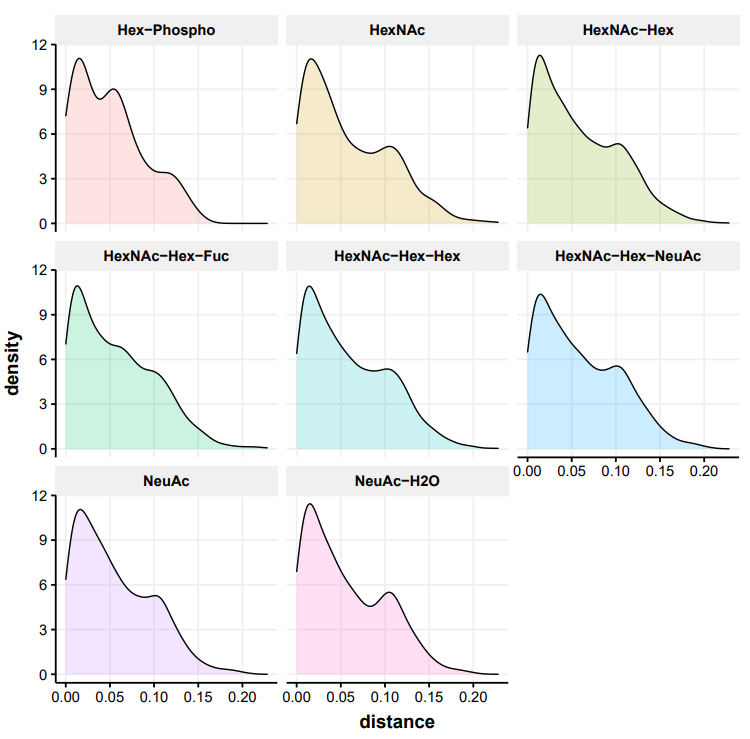


**Figure S8**. Distribution of the glyco-oxonium ion containing glycopeptides from human neutrophils inside the polygon demonstrating different glycopeptides with separate glycan types (high mannose vs sialylated) are indistinguishable by using the timsTOF Pro.


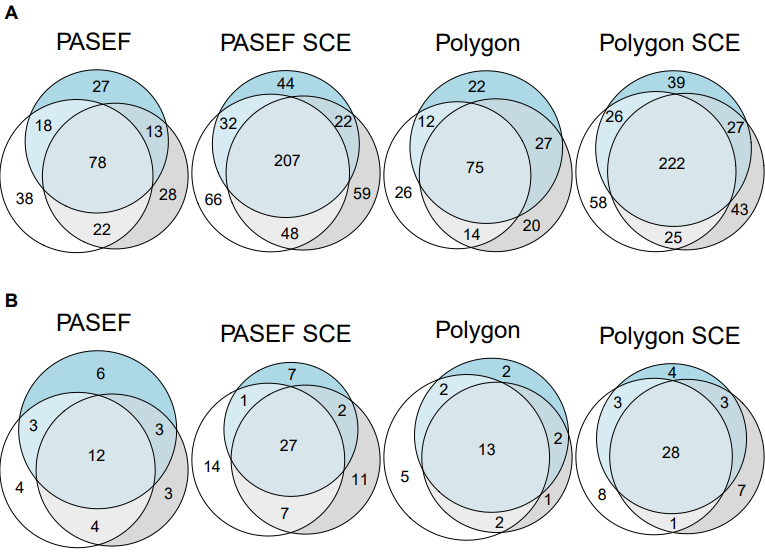


**Figure S9.** Performance comparison of four different methods on the timsTOF Pro for glycoproteomics applied to the neutrophil samples. Overlap of all the annotated **(A)** glycopeptides and **(B)** glycoproteins across three replicated MS/MS measurements in PASEF, PASEF SCE (*i.e* with stepped CE), PASEF with glyco-polygon and PASEF with glyco-polygon and stepped CE. Glyco-polygon demonstrates advantages on glycopeptide identification reproducibility within replicates.


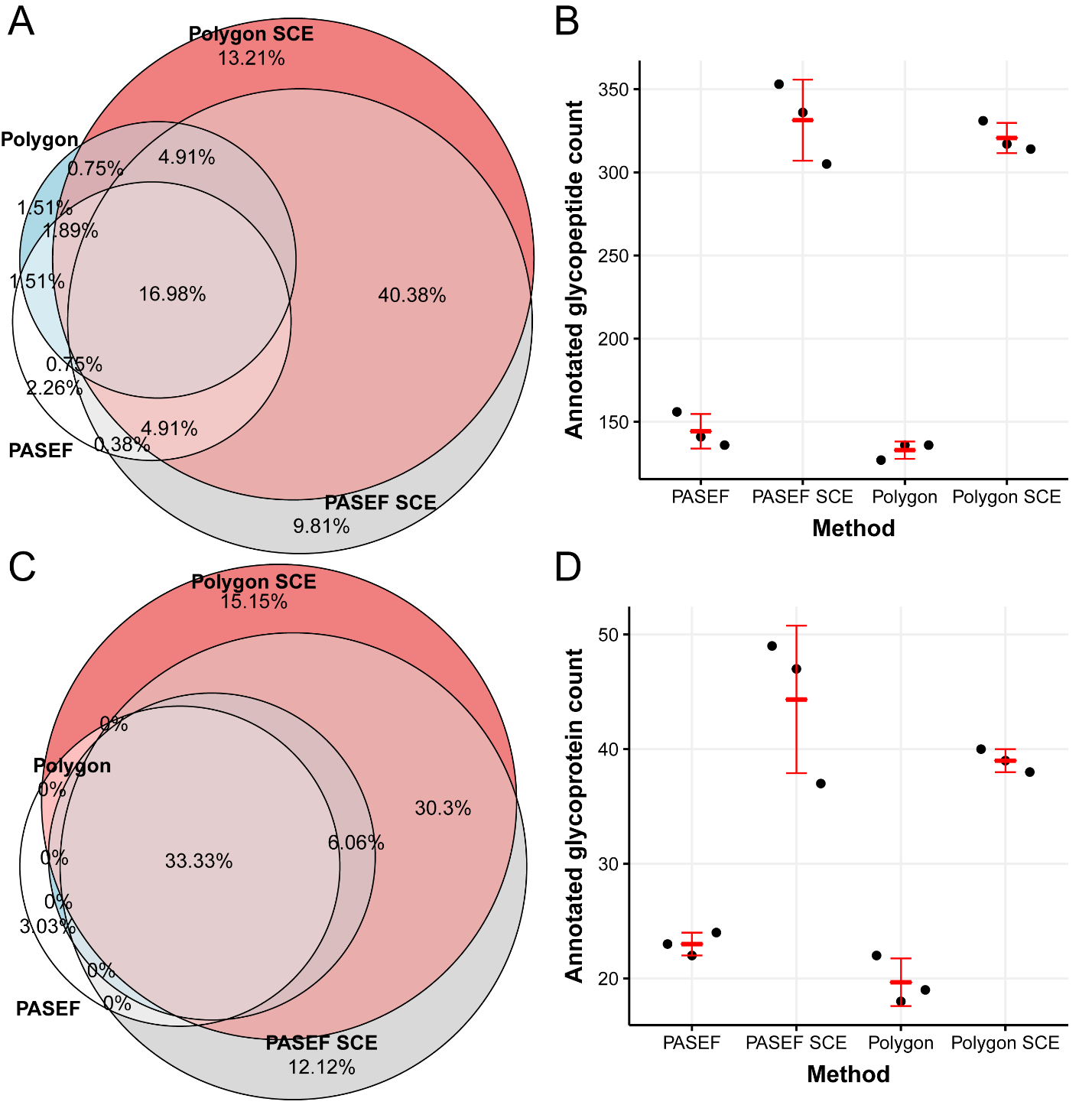


**Figure S10. Performance comparison of four different methods on the timsTOF Pro for glycoproteomics applied to the neutrophil samples**. Overlap of all the **(A)** glycopeptides, **(C)** glycoproteins annotated in all three technical replicates and counts of **(B)** annotated glycopeptides and **(D)** glycoproteins in PASEF, PASEF SCE (*i.e* with stepped CE), PASEF with glyco-polygon and PASEF with glyco-polygon and stepped CE. SCE method demonstrates clear advantages on glycopeptide identification while the IM polygon results in significant improvement of reproducibility across sample replicates.


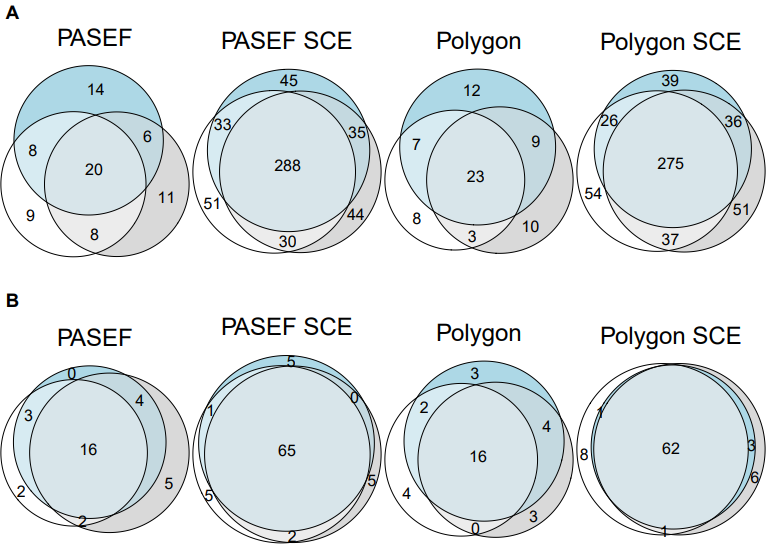


**Figure S11.** Performance comparison of four different methods on the timsTOF Pro for glycoproteomics applied to the plasma samples. Overlap of all the annotated **(A)** glycopeptides and **(B)** glycoproteins across three replicated MS/MS measurements in PASEF, PASEF SCE (*i.e.* with stepped CE), PASEF with glyco-polygon and PASEF with both glyco-polygon and stepped CE. Glyco-polygon demonstrates advantages on glycopeptide identification reproducibility within replicates.


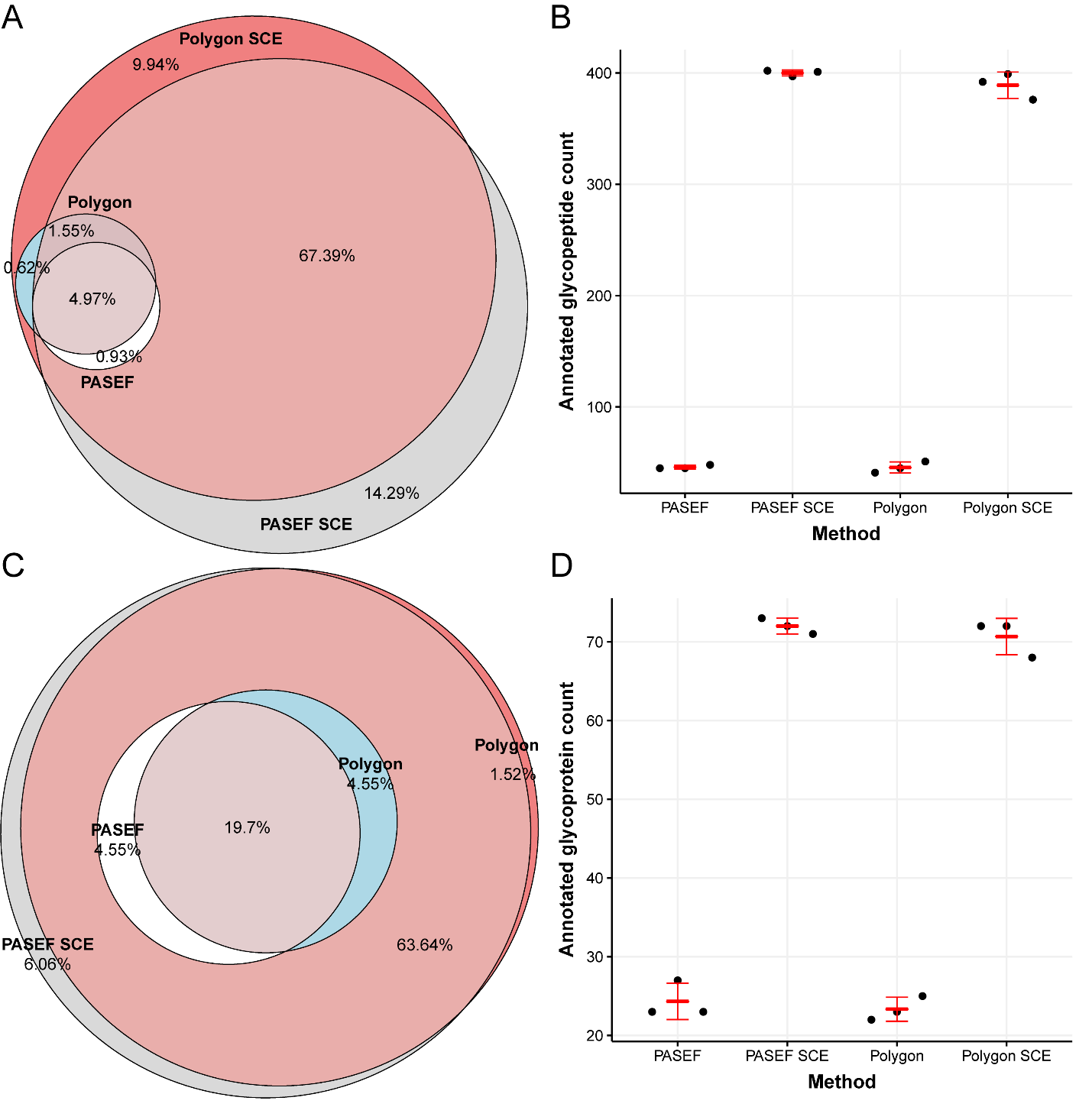


**Figure S12. Performance comparison of four different methods on the timsTOF Pro for glycoproteomics applied to the human plasma sample**. Overlap of all the **(A)** glycopeptides, **(C)** glycoproteins, **(B)** annotated glycopeptides and **(D)** glycoproteins in PASEF, PASEF (ST *i.e* with stepped CE), PASEF with glyco-polygon and PASEF with glyco-polygon and stepped CE. The use of the glyco-polygon demonstrates clear advantages on both glycopeptide identification and glycoprotein coverage.


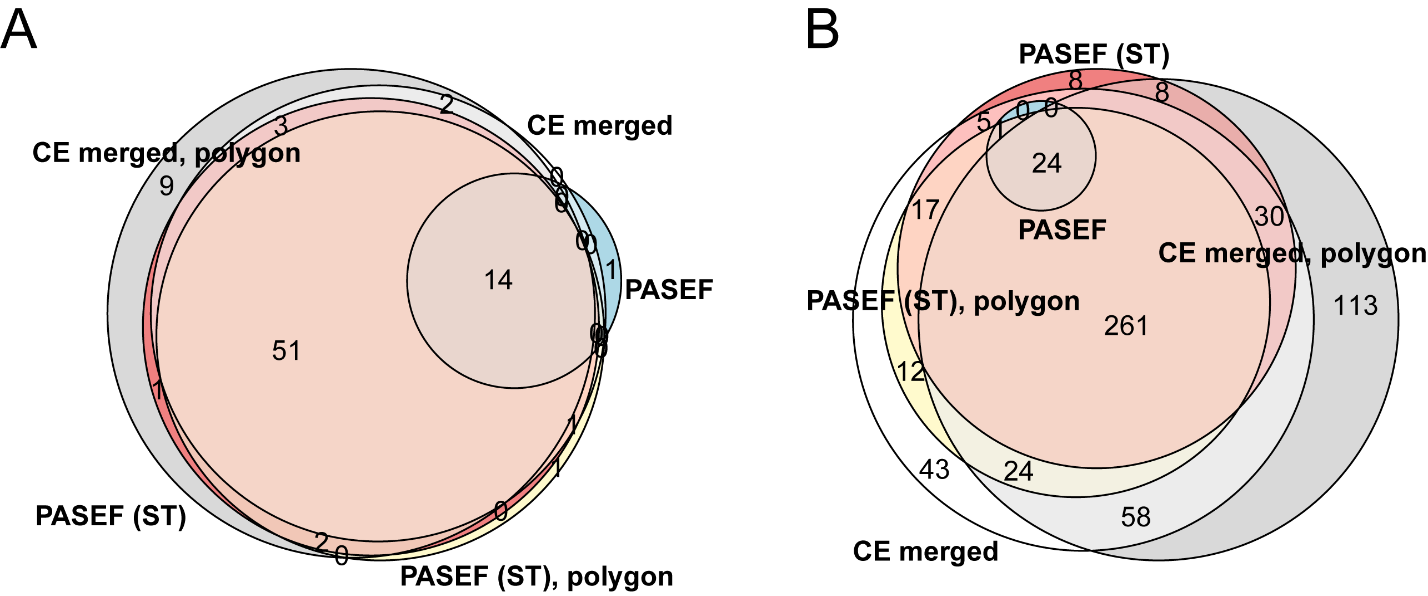


**Figure S13.** Performance comparison of five different methods on the timsTOF Pro for glycoproteomics applied to the human plasma sample. Overlap of all the annotated **(A)** glycoproteins and **(B)** glycopeptides demonstrate clear benefit of using more than two CE for precursor fragmentation during data acquisition.


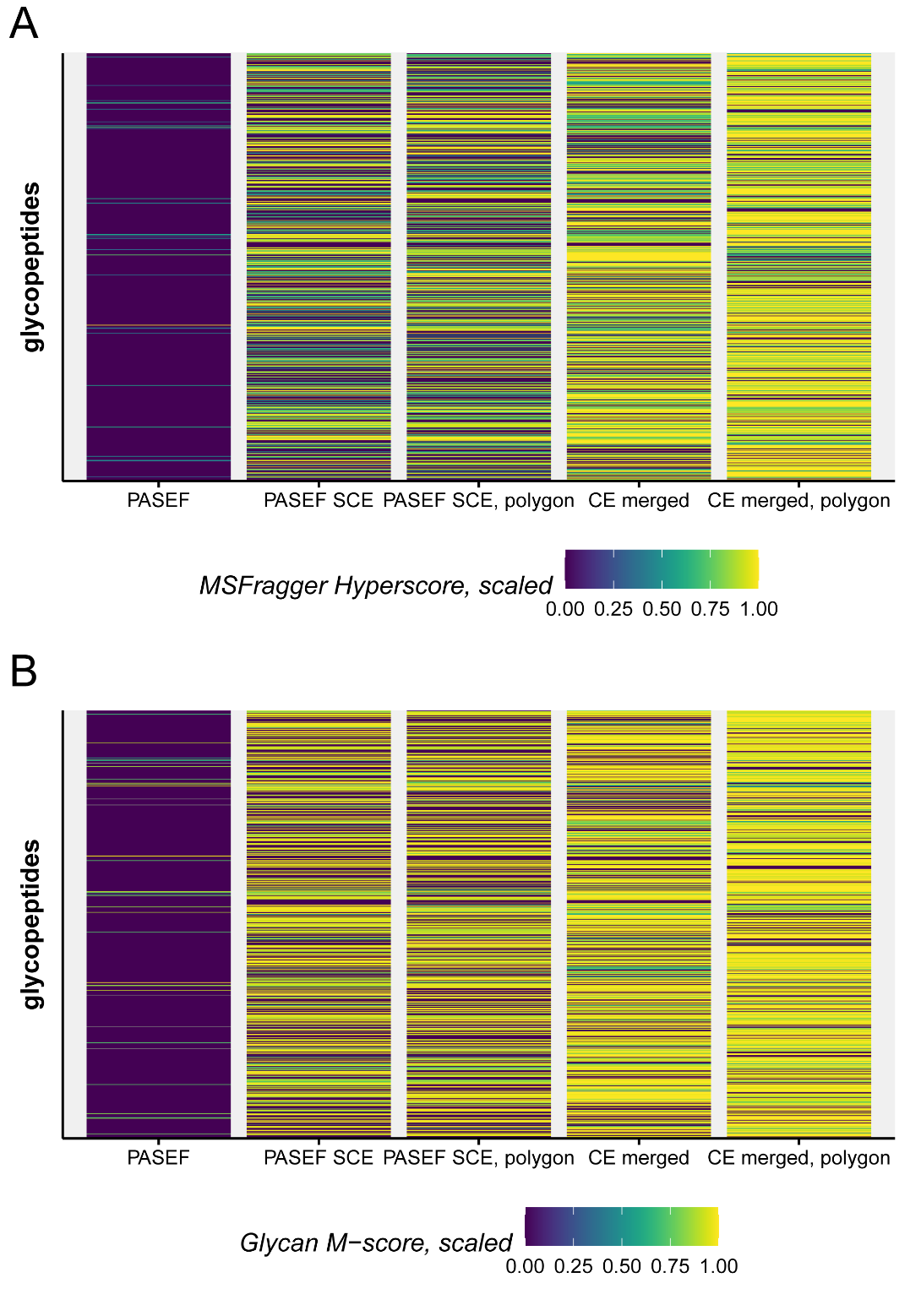


**Figure S14.** Performance comparison of five different methods on the timsTOF Pro for glycoproteomics applied to the human plasma sample. MSfragger peptide annotation score **(A)** and glycan M-score **(B)** both indicate clear increase in data files with CE merged spectra. Scores of each peptide from five different methods are scaled to the maximum value before plotting.


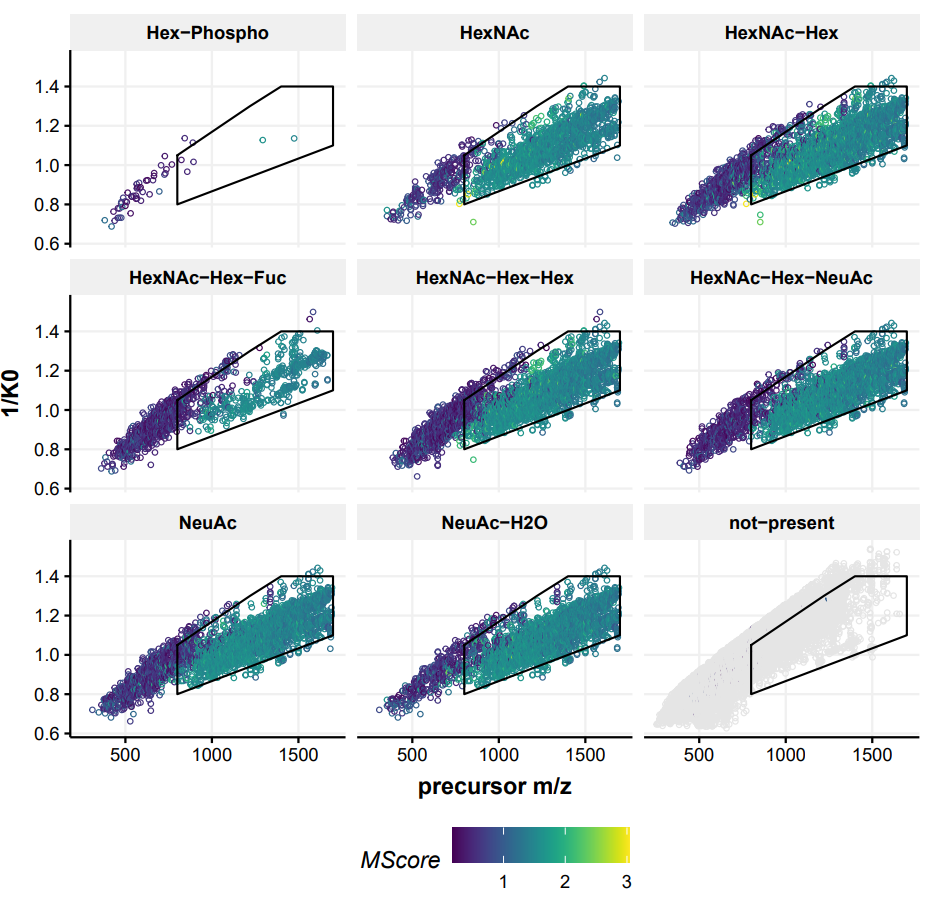


**Figure S15.** Density plot for the distribution for *m/z* vs reduced ion mobility (1/k_0_) of all the precursor ions containing the glyco-oxonium ions 243.0270 (Hex-Phospho), 204.0872 (HexNAc), 366.1400 (HexNAc-Hex), 512.198 (HexNAc-Hex-Fuc), 528.198 (HexNAc-Hex-Hex), 657.2354 (HexNAc-Hex-NeuAc), 292.1032 (NeuAc) and 274.0921 (NeuAc-H_2_O) from human plasma. The new stricter black polygon contains all the glycopeptide precursor with M-score > 1.3, describing the ROI for glycopeptide sequencing on the TIMSTOF Pro.


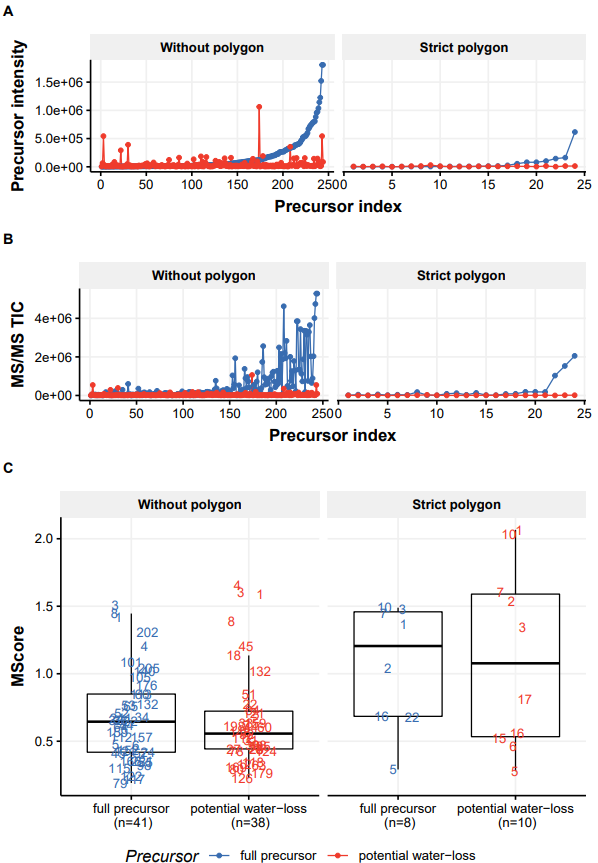


**Figure S16.** Distribution of potential water-loss precursors in human plasma sample with 90 minutes chromatographic gradient. Accurate mass of every precursor from the raw data file was matched against all other precursors for mass difference of water-loss fragment within 20 ppm mass and 20 seconds RT window. **(A)** Intensity of the pairs of matched precursors (blue) and its potential water-loss (red). Full precursors dominantly have a higher intensity thus expected to yield to higher quality fragmentation spectrum. **(B)** MS/MS spectrum total intensity of the pairs of matched precursors (blue) and its potential water-loss (red). Full precursors dominantly have a higher intensity thus expected to yield to higher quality fragmentation spectrum. **(C)** Distribution of glycan MScores for MS/MS spectra of the matched precursors where at least 2 oxonium ions have been observed. Numbers in the boxplot represent index of the matched precursor/water-loss pair.


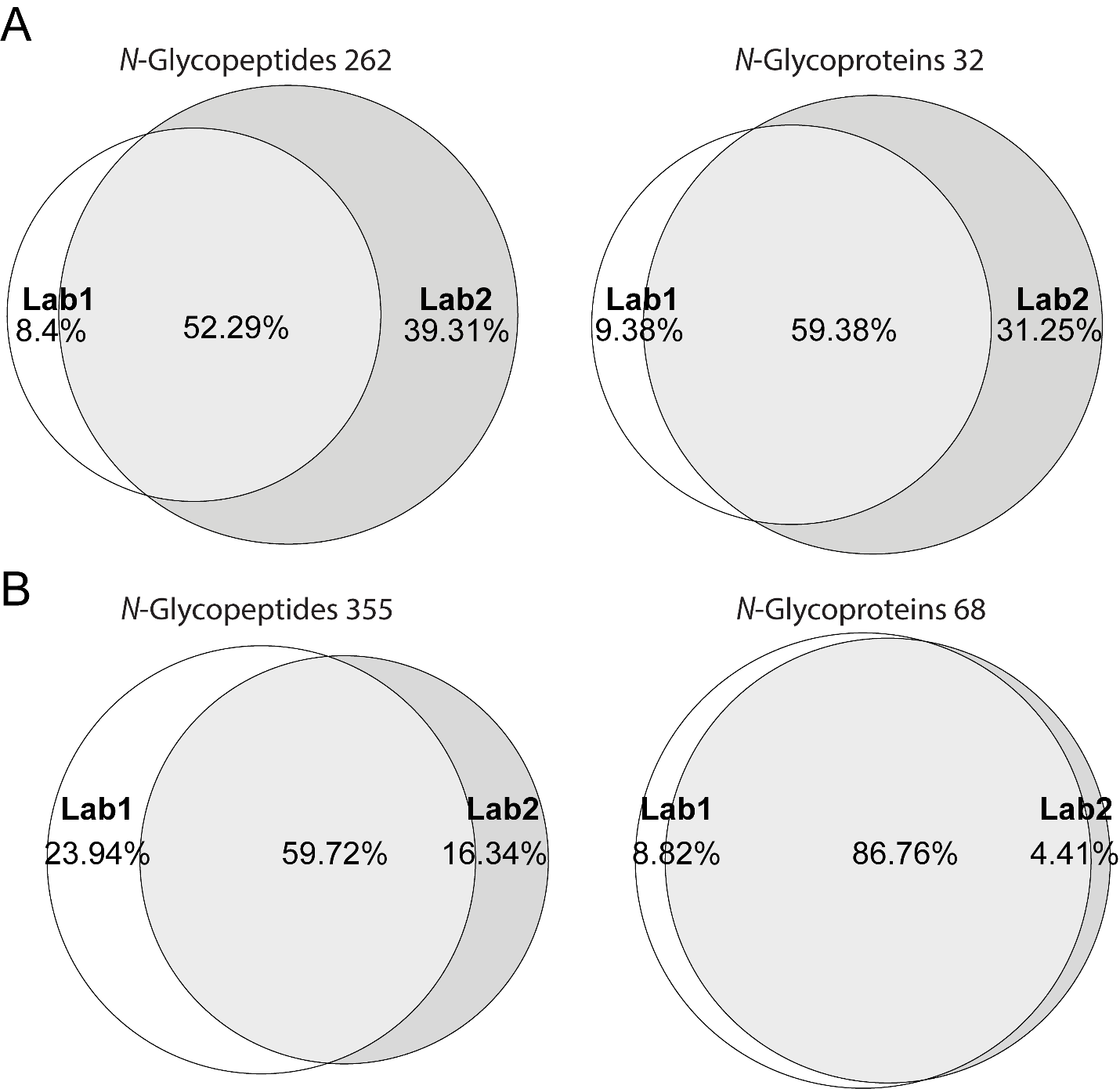


**Figure S17.** Interlaboratory comparison of the oxonium ion-guided ion mobility-assisted glycoproteomics workflow on the timsTOF Pro. Overlap of the *N*-glycopeptides and *N*-glycoproteins in the **(A)** human neutrophil and **(B)** human plasma sample that was shared between two different laboratories and analyzed using the SCE PASEF glyco-polygon method in triplicates. Only *N*-glycopeptides and *N*-glycoprotein present in all three replicates were used to compare the interlaboratory reproducibility.

**Table S1.** Gradient conditions (% B and time) on the columns (flow rate 400 nL/min) used in combinations with the optimized PASEF MS methods on the timsTOF Pro.

| % B (0.1 % FA, 99.9% ACN) | 150 min gradient (min) | 90 min gradient (min) | 60 min gradient (min) | 30 min gradient (min) | 15 min gradient (min) |
| --- | --- | --- | --- | --- | --- |
| 1 | 0 | 0 | 0 | 0 | 0 |
| 1 | 13.0 | 13.0 | 13.0 | 13.0 | 13.0 |
| 3 | 13.1 | 13.1 | 13.1 | 13.1 | 13.1 |
| 30 | 163.0 | 103.1 | 73.1 | 43.1 | 28.1 |
| 32 | 165.0 |  |  |  | - |
| 35 | - | 108.1 | 78 | 45.0 | 30.0 |
| 80 | 166.0 | 109 | 79 | 45.8 | 30.5 |
| 80 | 172.0 | 115 | 84 | 49.0 | 32.5 |
| 1 | 173.0 | 116 | 85 | 49.5 | 32.8 |
| 1 | 180.0 | 120 | 95 | 55.0 | 37.0 |

**Table S2**. Table of the MS parameters of the glyco polygon stepped collision energy (SCE) PASEF (Poylgon SCE PASEF), polygon PASEF (no SCE), SCE PASEF and strict plasma glyco specific polygon SCE PASEF compared to the original PASEF on the Bruker timsTOF pro that was used in this study.

| Parameters | PASEF | | SCE PASEF | | Polygon PASEF | | Polygon SCE PASEF | | | Plasma Polygon SCE PASEF | | |
| --- | --- | --- | --- | --- | --- | --- | --- | --- | --- | --- | --- | --- |
| MS/MS cycle | 10 | | 7 | | 10 | | 7 | | | 7 | | |
| PASEF cycle (s) | 1.16 | | 1.57 | | 1.16 | | 1.57 | | | 1.57 | | |
| *z* | 2-5 | | 2-5 | | 2-5 | | 2-5 | | | 2-5 | | |
| Intensity | 20,000 | | 20,000 | | 20,000 | | 20,000 | | | 20,000 | | |
| Intensity Threshold | 2500 | | 2500 | | 2500 | | 2500 | | | 2500 | | |
| Collison Energy (eV) | | | | | | | | | | | | |
| 1/k_0_ (V.s/cm^2^) | 0.6 | 1.6 | 0.5 | 1.6 | 0.6 | 1.6 | 0.5 | 1.6 | | 0.5 | 1.6 | |
| CE | 20 | 59 | 35 | 65 | 20 | 59 | 35 | 65 | | 35 | 65 | |
|  |  |  | 40 | 100 |  |  | 40 | 100 | | 40 | 100 | |
| Collision cell (Vpp) | 1500 V | | 1500 V | | 1500 V | | 1500 V | | | 1500 V | | |
| Pre-pulse storage time (µs) | 12 | | 12 | | 12 | | 12 | | | 12 | | |
| Transfer Time (µs) | 60 | | 60 | | 60 | | 60 | | | 60 | | |
| PASEF Polygon | 1/k_0_ (V.s/cm^2^) | *m/z* | 1/k_0_ (V.s/cm^2^) | *m/z* | 1/k_0_ (V.s/cm^2^) | *m/z* | 1/k_0_ (V.s/cm^2^) | | *m/z* | 1/k_0_ (V.s/cm^2^) | | *m/z* |
|  | 0.65 | 198 | 1.016 | 613 | 0.65 | 198 | 1.016 | | 613 | 1.05 | | 800 |
|  | 1.06 | 658.5 | 1.451 | 1222 | 1.06 | 658.5 | 1.451 | | 1222 | 1.30 | | 1220 |
|  | 1.24 | 954.3 | 1.605 | 1400 | 1.24 | 954.3 | 1.605 | | 1400 | 1.4 | | 1400 |
|  | 1.44 | 1263.5 | 1.605 | 1700 | 1.44 | 1263.5 | 1.605 | | 1700 | 1.4 | | 1700 |
|  | 1.58 | 1418.6 | 0.798 | 1700 | 1.58 | 1418.6 | 0.798 | | 1700 | 1.10 | | 1700 |
|  | 1.58 | 1715.3 | - | - | 1.58 | 1715.3 | - | | - | 0.80 | | 800 |
|  | 0.62 | 1715.3 | - | - | 0.62 | 1715.3 | - | | - | - | | - |
|  | 0.61 | 194.2 | - | - | 0.61 | 194.2 | - | | - | - | | - |

**Table S3**. **Table layout of the method files used for interlaboratory comparison for both MSfragger and Byonic database search. Glyco-polygon SCE-PASEF was MS method used.**

| File Names | Sample | Method | Figure | Output Files | Laboratory |
| --- | --- | --- | --- | --- | --- |
| 250ng_Neutrophil_digest_d_PASEF_Polygon_Stepped_Method_Slot1-1_1_7862.d | Human neutrophils | Polygon SCE PASEF | Fig 7; Fig. 8; Fig. S7-S8 | Byonic_curated_output_Excel.xlsx; Human_Neutrophil_all_psms.tsv (Msfragger output) | Laboratory 2 |
| 250ng_Neutrophil_digest_d_PASEF_Polygon_Stepped_Method_Slot1-1_1_7867.d | Human neutrophils | Polygon SCE PASEF | Fig 7; Fig. 8; Fig. S7-S8 | Byonic_curated_output_Excel.xlsx; Human_Neutrophil_all_psms.tsv (Msfragger output) |  |
| 250ng_Neutrophil_digest_d_PASEF_Polygon_Stepped_Method_Slot1-1_1_7872.d | Human neutrophils | Polygon SCE PASEF | Fig 7; Fig. 8; Fig. S7-S8 | Byonic_curated_output_Excel.xlsx; Human_Neutrophil_all_psms.tsv (Msfragger output) |  |
| 20220224_TIMSTOF1_UM6_Mukhe004_SA_EXT00_HumanNeutrophil250ng_PASEF_Polygon_Stepped_RA11_1_1584.d | Human neutrophils | Polygon SCE PASEF | Fig. 7; Fig. 8; Fig. S16 | Byonic_curated_output_Excel.xlsx | Laboratory 1 |
| 20220228_TIMSTOF1_UM6_Mukhe004_EXT00_HumanNeutrophil250ng_Polygon_stepped_R3_RA11_1_1652.d | Human neutrophils | Polygon SCE PASEF | Fig. 7; Fig. 8; Fig. S16 | Byonic_curated_output_Excel.xlsx |  |
| 20220228_TIMSTOF1_UM6_Mukhe004_EXT00_HumanNeutrophil250ng_Polygon_stepped_R2_RA11_1_1651.d | Human neutrophils | Polygon SCE PASEF | Fig. 7; Fig. 8; Fig. S16 | Byonic_curated_output_Excel.xlsx |  |
| 20220228_TIMSTOF1_UM6_Mukhe004_EXT00_HumanPlasma250ng_Polygon_stepped_150min_R1_RA12_1_1633.d | Human Plasma | Polygon SCE PASEF | Fig S7-8 | Msfragger output | Laboratory 1 |
| 20220228_TIMSTOF1_UM6_Mukhe004_EXT00_HumanPlasma250ng_Polygon_stepped_150min_R2_RA12_1_1634.d | Human Plasma | Polygon SCE PASEF | Fig. S7-8 | Msfragger output |  |
| 20220228_TIMSTOF1_UM6_Mukhe004_EXT00_HumanPlasma250ng_Polygon_stepped_150min_R3_RA12_1_1635.d | Human Plasma | Polygon SCE PASEF | Figure S7-8 | Msfragger output |  |
| 250ng_Plasma_digest_d_PASEF_Polygon_Stepped_Method_Slot1-2_1_7877.d | Human plasma | Polygon SCE PASEF | Fig 7; Fig. 8; Fig. S7-S8 | Byonic_curated_output_Excel.xlsx; Human_plasma_all_psms.tsv | Laboratory 2 |
| 250ng_Plasma_digest_d_PASEF_Polygon_Stepped_Method_Slot1-2_1_7882.d | Human plasma | Polygon SCE PASEF | Fig 7; Fig. 8; Fig. S7-S8 | Byonic_curated_output_Excel.xlsx; Human_plasma_all_psms.tsv |  |
| 250ng_Plasma_digest_d_PASEF_Polygon_Stepped_Method_Slot1-2_1_7887.d | Human plasma | Polygon SCE PASEF | Fig 7; Fig. 8; Fig. S7-S8 | Byonic_curated_output_Excel.xlsx; Human_plasma_all_psms.tsv |  |
| 20220322_TIMSTOF1_UM6_Mukhe004_EXT00_Plasma250ng_PolygonPlasma_Stepped_90min_R1_RD2_1_1695.d | Human plasma | PASEF SCE, Polygon | Fig. 6 (E, F); Fig. 7; Fig. 8; Fig. S15 | Human_plasma_RT_gradients_psm.tsv; Byonic_curated_output_Excel.xlsx | Laboratory 1 |
| 20220322_TIMSTOF1_UM6_Mukhe004_EXT00_Plasma250ng_PolygonPlasma_Stepped_90min_R2_RD2_1_1696.d | Human plasma | PASEF SCE, Polygon | Fig. 6 (E, F); Fig. 7; Fig. 8; Fig. S15 | Human_plasma_RT_gradients_psm.tsv; Byonic_curated_output_Excel.xlsx |  |
| 20220322_TIMSTOF1_UM6_Mukhe004_EXT00_Plasma250ng_PolygonPlasma_Stepped_90min_R3_RD2_1_1697.d | Human plasma | PASEF SCE, Polygon | Fig. 6 (E, F); Fig. 7; Fig. 8; Fig. S15 | Human_plasma_RT_gradients_psm.tsv; Byonic_curated_output_Excel.xlsx |  |

**Table S4.** **Monosaccharide identities and exact masses of the glyco-oxonium ions.**

| **Mass** (m/z) | **Chemical Formula** | **Glycan** |
| --- | --- | --- |
| 204.087199 | [H14C8N1O5]^+^ | HexNAc |
| 274.0921 | [H16C11N1O7]^+^ | NeuAc-H2O |
| 292.103244 | H18C11N1O8]^+^ | NeuAc |
| 366.140024 | H24C14N1O10]^+^ | HexNAc-Hex |
| 657.235443 | H41C25N2O18]^+^ | HexNAc-Hex-NeuAc |
| 528.192849 | H34C20N1O15]^+^ | HexNAc-Hex-Hex |
| 512.197933 | H34C20N1O14]^+^ | HexAc-Hex-Fuc |
| 243.026983 | H12C6O8P1]^+^ | Hex-Phospho |

**Table S5. Performance comparison of different methods of the timsTOF Pro for glycoproteomics applied to the human neutrophil sample.**

| **Method** | **Replicate** | **Glyco psms** | **Unique glycopeptides** | **Unique glycoproteins** |
| --- | --- | --- | --- | --- |
| PASEF | 1 | 247 | 156 | 23 |
| PASEF | 2 | 219 | 141 | 22 |
| PASEF | 3 | 205 | 136 | 24 |
| PASEF polygon | 1 | 216 | 127 | 22 |
| PASEF polygon | 2 | 225 | 136 | 18 |
| PASEF polygon | 3 | 206 | 136 | 19 |
| SCE-PASEF | 1 | 609 | 353 | 49 |
| SCE-PASEF | 2 | 570 | 336 | 47 |
| SCE-PASEF | 3 | 512 | 305 | 37 |
| SCE-PASEF polygon | 1 | 590 | 331 | 40 |
| SCE-PASEF polygon | 2 | 541 | 317 | 39 |
| SCE-PASEF polygon | 3 | 531 | 314 | 38 |

**Table S6. Performance comparison of different methods of the timsTOF Pro for glycoproteomics applied to the human plasma sample.**

| **Method** | **Replicate** | **Glyco psms** | **Unique glycopeptides** | **Unique glycoproteins** |
| --- | --- | --- | --- | --- |
| PASEF | 1 | 77 | 45 | 23 |
| PASEF | 2 | 76 | 45 | 27 |
| PASEF | 3 | 80 | 48 | 23 |
| PASEF polygon | 1 | 74 | 41 | 22 |
| PASEF polygon | 2 | 79 | 45 | 23 |
| PASEF polygon | 3 | 97 | 51 | 25 |
| SCE-PASEF | 1 | 895 | 402 | 73 |
| SCE-PASEF | 2 | 868 | 397 | 72 |
| SCE-PASEF | 3 | 872 | 401 | 71 |
| SCE-PASEF polygon | 1 | 889 | 392 | 72 |
| SCE-PASEF polygon | 2 | 874 | 399 | 72 |
| SCE-PASEF polygon | 3 | 844 | 376 | 68 |

**Table S7.Performance comparison of different methods and CE spectra summing on the timsTOF Pro for glycoproteomics applied to the human plasma sample**.

| **Method** | **Glyco psms** | **Unique glycopeptides** | **Unique glycoproteins** |
| --- | --- | --- | --- |
| PASEF | 51 | 29 | 15 |
| SCE-PASEF | 788 | 368 | 72 |
| SCE-PASEF, polygon | 820 | 378 | 69 |
| CE merged | 1052 | 478 | 71 |
| CE polygon merged | 1259 | 545 | 82 |

**Table S8. Performance comparison of SCE-PASEF method with/without strict glyco-polygon of the human plasma sample with different chromatography gradients.**

| **Method, LC gradient length, min** | **Replicate** | **Glyco psms** | **Unique glycopeptides** | **Unique glycoproteins** |
| --- | --- | --- | --- | --- |
| Without polygon, 15 | 1 | 97 | 77 | 32 |
| Without polygon, 15 | 2 | 92 | 69 | 28 |
| Without polygon, 15 | 3 | 99 | 73 | 25 |
| Without polygon, 30 | 1 | 276 | 185 | 51 |
| Without polygon, 30 | 2 | 285 | 189 | 48 |
| Without polygon, 30 | 3 | 275 | 183 | 47 |
| Without polygon, 60 | 1 | 574 | 332 | 65 |
| Without polygon, 60 | 2 | 560 | 328 | 63 |
| Without polygon, 60 | 3 | 582 | 348 | 67 |
| Without polygon, 90 | 1 | 667 | 376 | 67 |
| Without polygon, 90 | 2 | 678 | 367 | 67 |
| Without polygon, 90 | 3 | 700 | 384 | 69 |
| Polygon, 15 | 1 | 162 | 112 | 37 |
| Polygon, 15 | 2 | 160 | 108 | 36 |
| Polygon, 15 | 3 | 155 | 110 | 36 |
| Polygon, 30 | 1 | 438 | 271 | 63 |
| Polygon, 30 | 2 | 441 | 276 | 57 |
| Polygon, 30 | 3 | 470 | 273 | 55 |
| Polygon, 60 | 1 | 732 | 381 | 69 |
| Polygon, 60 | 2 | 758 | 393 | 74 |
| Polygon, 60 | 3 | 729 | 388 | 68 |
| Polygon, 90 | 1 | 873 | 454 | 74 |
| Polygon, 90 | 2 | 893 | 451 | 74 |
| Polygon, 90 | 3 | 905 | 451 | 74 |
